# Curcuminoid Binding to Embryonal Carcinoma Cells: Reductive Metabolism, Induction of Apoptosis, Senescence, and Inhibition of Cell Proliferation

**DOI:** 10.1371/journal.pone.0039568

**Published:** 2012-06-26

**Authors:** Wolfgang W. Quitschke

**Affiliations:** Department of Psychiatry and Behavioral Science, State University of New York at Stony Brook, Stony Brook, New York, United States of America; City of Hope, United States of America

## Abstract

Curcumin preparations typically contain a mixture of polyphenols, collectively referred to as curcuminoids. In addition to the primary component curcumin, they also contain smaller amounts of the co-extracted derivatives demethoxycurcumin and bisdemethoxycurcumin. Curcuminoids can be differentially solubilized in serum, which allows for the systematic analysis of concentration-dependent cellular binding, biological effects, and metabolism. Technical grade curcumin was solubilized in fetal calf serum by two alternative methods yielding saturated preparations containing either predominantly curcumin (60%) or bisdemethoxycurcumin (55%). Continual exposure of NT2/D1 cells for 4–6 days to either preparation in cell culture media reduced cell division (1–5 µM), induced senescence (6–7 µM) or comprehensive cell death (8–10 µM) in a concentration-dependent manner. Some of these effects could also be elicited in cells transiently exposed to higher concentrations of curcuminoids (47 µM) for 0.5–4 h. Curcuminoids induced apoptosis by generalized activation of caspases but without nucleosomal fragmentation. The equilibrium binding of serum-solubilized curcuminoids to NT2/D1 cells incubated with increasing amounts of curcuminoid-saturated serum occurred with apparent overall dissociation constants in the 6–10 µM range. However, the presence of excess free serum decreased cellular binding in a hyperbolic manner. Cellular binding was overwhelmingly associated with membrane fractions and bound curcuminoids were metabolized in NT2/D1 cells via a previously unidentified reduction pathway. Both the binding affinities for curcuminoids and their reductive metabolic pathways varied in other cell lines. These results suggest that curcuminoids interact with cellular binding sites, thereby activating signal transduction pathways that initiate a variety of biological responses. The dose-dependent effects of these responses further imply that distinct cellular pathways are sequentially activated and that this activation is dependent on the affinity of curcuminoids for the respective binding sites. Defined serum-solubilized curcuminoids used in cell culture media are thus suitable for further investigating the differential activation of signal transduction pathways.

## Introduction

Curcumin has been implicated as beneficial in numerous medicinal applications. These include inhibition of tumor propagation, protection against Alzheimer disease and antiinflammatory properties. *In vitro*, curcumin slows the rate of cell division or induces apoptosis in a concentration-dependent manner (for reviews see: [1], [2], [3], [4], [5]).

Curcuminoids are polyphenols extracted from the root of Curcuma longa [6], [7]. They impart the yellow color to the spice turmeric, where they occur in amounts of 2–8% [8], [9], [10]. Curcumin is the main curcuminoid ingredient in turmeric (65–80%), in addition to smaller amounts of two structurally related derivatives that lack either one (demethoxycurcumin, 15–25%) or both (bisdemethoxycurcumin, 5–15%) methoxy groups in the phenyl rings [11], [12]. Collectively, these compounds are referred to as curcuminoids, and they are typically co-extracted from turmeric in commercial preparations of curcumin. All curcuminoids have been implicated in biological activity, although the extent of their relative contributions may differ [2].

Curcuminoids are highly insoluble in aqueous solutions. Therefore, in cell culture studies, small amounts of a concentrated stock solution of curcuminoids predissolved in an organic solvent are typically added to the cell culture medium to achieve desired final concentrations. However, upon adding such predissolved curcuminoids to aqueous solutions, they initially precipitate. Although some or most of the precipitated material may gradually dissolve, the final concentrations and compositions of the actual soluble curcuminoids remain inferential, since they are based on the dilution of the total amount added. Methods have been described that differentially solubilize high concentrations of biologically active curcuminoids directly in serum. This provides an opportunity to prepare tissue culture media containing defined concentrations of serum-solubilized curcuminoids with varying compositions [13].

The effect of such curcuminoid preparations on NT2/D1 embryonal carcinoma cells was here systematically investigated in terms of binding, metabolism, and biological effects. These cells divide rapidly with limited contact inhibition and they are also capable of differentiating into a variety of neuronal and non-neuronal cell types, depending on culture conditions and upon exposure to specific inducers such as retinoic acid and hexamethylene bisacetamide [14]. This cell line has also been used to investigate the effect of other anticancer drugs [15], [16] and mechanisms of developmental processes [17]. Some results were compared to those obtained with HeLa and CCF-STTG1 cells.

## Results

### Curcuminoids Solubilized in Fetal Calf Serum Differentially Bind to NT2/D1 Cells

**Figure 1:**
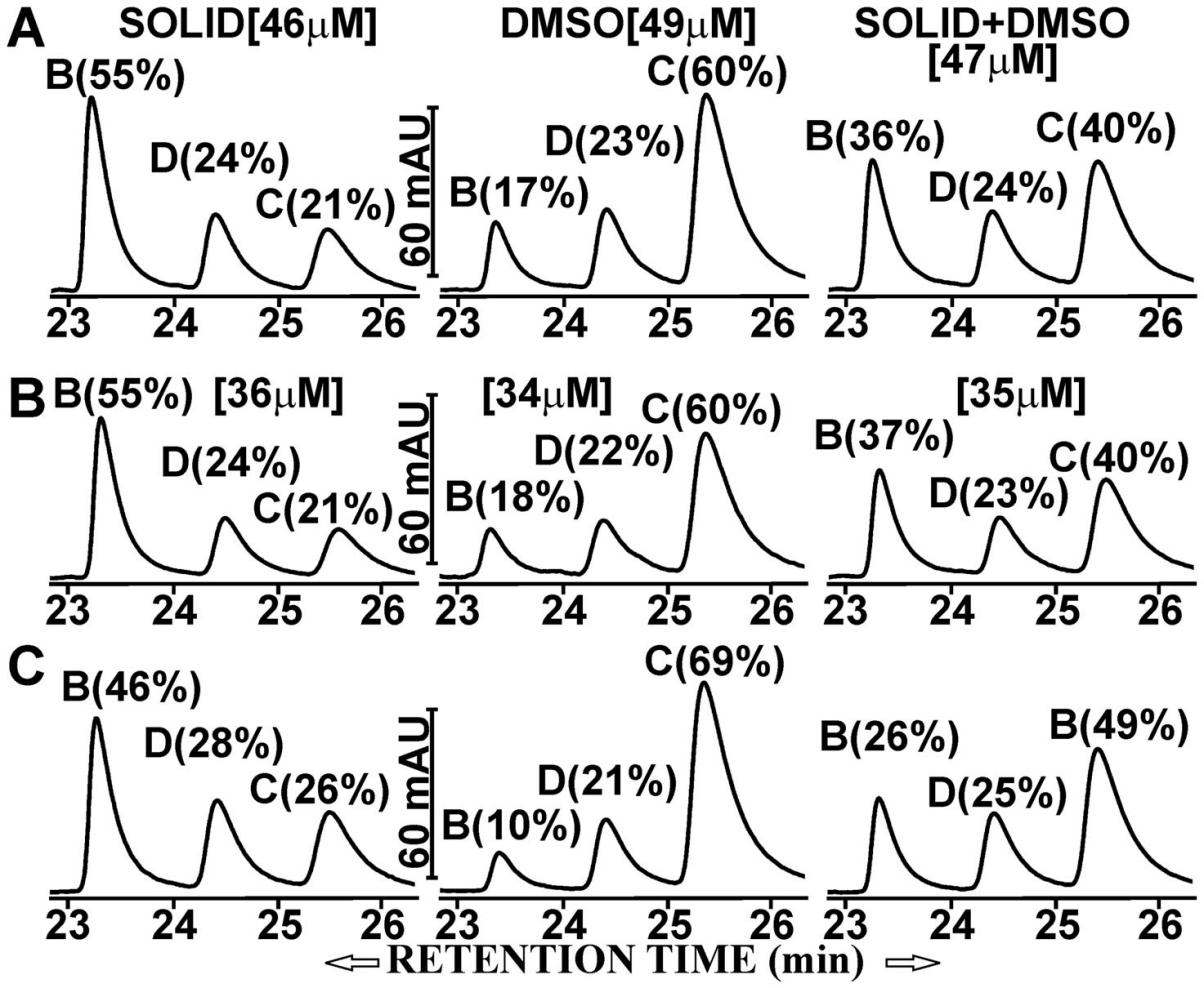
Distribution of curcuminoids in NT2/D1 cells and culture medium. Left panel: SOLID-solubilized curcuminoids in FCS (SOLID). Center panel: DMSO-solubilized curcuminoids in FCS (DMSO). Right panel: A 1∶1 mixture of SOLID- and DMSO-solubilized curcuminoids (SOLID+DMSO). Square brackets show total curcuminoid concentrations in media. The relative molar amounts (%) of BDMC (B), DMC (D), and CUR (C) are indicated in each panel. (A) Starting composition of curcuminoids in media before incubation with cells. (B). Curcuminoid composition in media after 1 h incubation with cells. (C) Curcuminoids extracted from cells after 1 h incubation with media depicted in A.

The curcumin preparation used in this study (technical grade, Cayman Chemical Company) contained a mixture of curcumin (CUR, 69%), demethoxycurcumin (DMC, 19%), and bisdemethoxycurcumin (BDMC, 12%). These curcuminoids were solubilized in fetal calf serum (FCS) either by mixing them directly with the solid powder (SOLID-solubilized) or by adding small aliquots of curcuminoids dissolved in dimethylsulfoxide (DMSO-solubilized). This procedure yielded curcuminoid-saturated preparations, that could be further diluted with cell culture media without precipitations or changes in composition [13]. Both preparations were directly diluted with cell culture medium (DMEM, Dulbecco’s Minimal Essential Medium) for a final concentration of 5% FCS. Although the total amount of solubilized curcuminoids was similar (46–49 µM), their composition differed between the two methods of preparation. In medium containing serum with SOLID-solubilized curcuminoids, BDMC (55%) was preferentially solubilized with smaller amounts of DMC (24%) and CUR (21%). By comparison, media containing serum with DMSO-solubilized curcuminoids were composed primarily of CUR (60%) with smaller amounts of DMC (23%) and BDMC (17%), which is similar to the curcuminoid content of the original powdered preparation. A mixture (1∶1) of the two preparations (SOLID+DMSO) produced a more balanced curcuminoid composition (CUR: 40%, DMC: 24%, and BDMC: 36%) (Fig. 1A).

NT2/D1 cells were incubated for 1 h with media containing these three curcuminoid preparations (SOLID, DMSO, SOLID+DMSO) in a total FCS concentration of 5%. The amount of curcuminoids bound to cells and their concentrations in the media were then determined. In all cases, the total curcuminoid concentrations in the media had declined to levels of 70–75% of the original value while the overall composition of curcuminoids remained largely unchanged (Fig. 1B). A significant amount of curcuminoids remained bound to the NT2/D1 cells. However, the cellular-bound curcuminoids showed different compositions than those found in the media. In general, the relative amount of cellular-bound CUR had increased at the expense of BDMC while the variation in the relative amount of DMC was less consistent (Fig. 1C). These observations served as the basis for further experiments in this study.

### Curcuminoids Reduce NT2/D1 Proliferation Rates or Induce Cell Death in a Dose-dependent Manner

**Figure 2:**
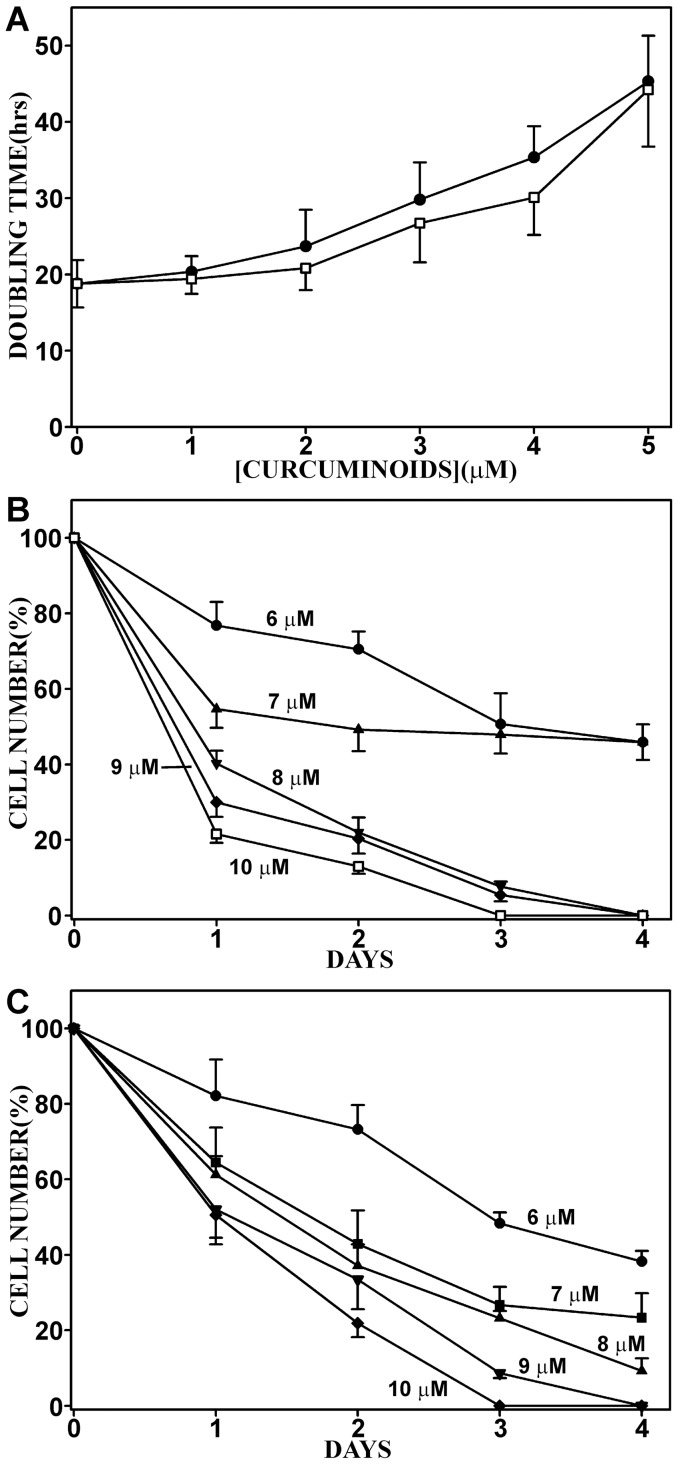
Concentration-dependent effects of curcuminoids on NT2/D1 cell division and survival (A) The effect of 0–5 µM curcuminoid starting concentrations in incubation media on cellular doubling time. Curcuminoids were either SOLID-(□) or DMSO-solubilized (▪). (B) The effect on cell survival of 6–10 µM starting concentrations of SOLID-solubilized curcuminoids. (C) The effect on cell survival of 6–10 µM DMSO-solubilized curcuminoids.

The variation in relative solubility of SOLID- and DMSO-solubilized curcuminoids in FCS and their concomitant binding to NT2/D1 cells allows for a comparison of their possible differential biological effects. To assess the influence of curcuminoids on cell proliferation and survival, media containing saturated concentrations of SOLID-(46 µM) and DMSO-solubilized (49 µM) were diluted with standard medium (DMEM, 5% FCS without curcuminoids) to attain 1–10 µM curcuminoid concentrations. NT2/D1 cells at 20–30% confluence were then incubated for 4–6 days and the media were replenished every 24 h. A dose-dependent reduction in the rate of cell division was observed at 1–5 µM curcuminoid concentrations. The cellular doubling time gradually increased from about 19 h in cultures grown without curcuminoids, to 44 h in media containing 5 µM initial curcuminoid concentrations. There was no evidence of significant cell death at these curcuminoid concentrations and the rate of cell growth was similar in cells exposed to preparations containing either SOLID- or DMSO-solubilized curcuminoids (Fig. 2A). At 6–7 µM curcuminoid concentrations, partial cell death was apparent within the first two days of incubation. However, the cell number thereafter stabilized at a level of 40–60% of the starting density. Further cell division was suppressed and the surviving cells remained viable while undergoing changes in morphology (see below). At higher 8–10 µM curcuminoid concentrations, dose-dependent cell death was evident with no surviving cells after four days of incubation. No significant differences were noted in the cellular responses between SOLID- and DMSO-solubilized curcuminoid preparations (Fig. 2B, C). By comparison, HeLa and CCF-STTG1 cells were considerably less sensitive to the effects of curcuminoids and required up to 3-fold higher minimal concentrations to induce comprehensive cell death (Table 1).

**Table 1:** Selected curcuminoid effects and binding parameters in different cell lines.

	NT2/D1	HELA	CCF-STTG1
Doubling time (S.D.)^1^	18.94 (0.74) h	27.81 (3.57) h	35.53 (2.85) h
K_D_-SOLID (S.E.)	6.3 (1.0) µM	17.0 (2.9) µM	25.6 (5.2) µM
B_MAX_-SOLID (S.E.)^2^	19.0 (0.8) nmol	12.5 (1.3) nmol	15.7 (1.5) nmol
K_D_-DMSO (S.E)	7.2 (1.5) µM	22.2 (3.5) µM	29.9 (7.5) µM
B_MAX_-DMSO (S.E.)^2^	22.0 (1.2) nmol	15.8 (1.2) nmol	20.4 (2.7) nmol
Cell Death [Curcuminoids]^3^	≥8 µM	≥20 µM	≥24 µM
Senescence [Curcuminoids]^3^	6–7 µM	18–19 µM	20–22 µM
Primary Metabolites (after 24 h incubation)	C*, D*, B* 4	Hexahydro-curcuminoids	Hexahydro-Octahydro-curcuminoids

1 Doubling time with standard deviation (S.D.) of cells grown in DMEM with 5% fetal calf serum.

2 Maximum specific cellular binding of curcuminoids (B_MAX_) determined from the respective binding curves and presented as the amount bound to cells (nmol) per mg of total cellular protein with standard error (S.E.).

3 Determined with SOLID-solubilized curcuminoids. The values indicate the required minimal initial concentrations of curcuminoids in the media to achieve 100% cell death within 4 d of incubation with media changes every 24 h. Cellular senescence was assessed after 6 days of incubation.

4 1,7-bis(4-hydroxy-3-methoxyphenyl)-5-hydroxy-1-heptene-3-one [CUR metabolite, C*]. 1-(4-hydroxy-3-methoxyphenyl)-7-(4-hydroxyphenyl)-5-hydroxy-1-heptene-3-one [DMC metabolite, D*]. 1,7-bis (4-hydroxyphenyl)-5-hydroxy-1-heptene-3-one [BDMC metabolite, B*].

### Cellular Senescence of NT2/D1 Cells in Response to Curcuminoids

**Figure 3:**
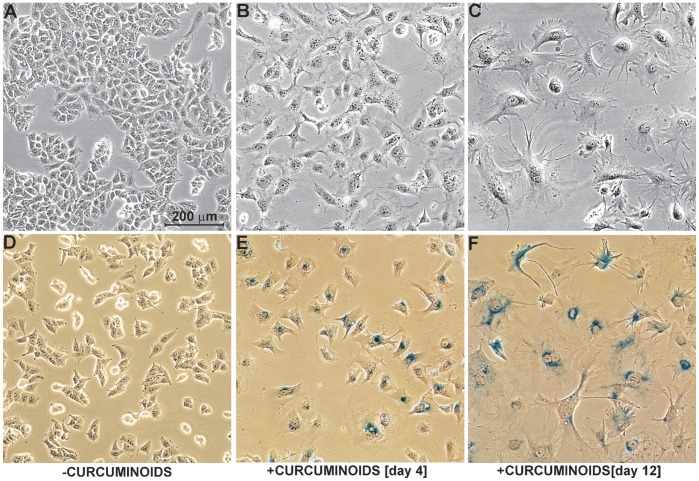
Cellular senescence in response to curcuminoids. Phase contrast micrographs of NT2/D1 cells incubated in media without curcuminoids (A and D) or with 6 µM SOLID-solubilized curcuminoids in FCS for 4 (B and E) and 12 (C and F) days. Panels D, E, and F show cells stained for SA-β-galactosidase activity at pH 6 (blue color).

The cellular response of NT2/D1 cells at intermediate 6–7 µM curcuminoid concentrations was further examined by phase contrast microscopy. NT2/D1 cells grown in standard medium were ordinarily relatively small and tended to propagate in coalescing groups (Fig 3A). However, soon after incubation with 6–7 µM curcuminoids, cell division stopped and limited cell death was apparent. After the first 4 days of incubation, the remaining individual cells had grown larger and distributed themselves over the available growth surface (Fig. 3B). After 12 days, individual cells had further expanded in size, concurrent with a gradual but limited decrease in cell number. Profound changes in cellular morphology were apparent with increased cytoplasmic, nuclear, and nucleolar size. The cytoplasm was often vacuolated with numerous irregular processes and polynucleated cells were frequently observed (Fig 3C). These changes were irreversible in that they persisted for weeks even after curcuminoid-containing media had been replaced with standard media. These terminal morphological changes were reminiscent of cells undergoing senescence [18], [19]. To further evaluate this possibility, dividing cells in standard media and cells exposed to 6–7 µM curcuminoids were stained for the presence of SA-β-galactosidase [20]. The detection of this compound at suboptimal pH conditions (pH 6) reflects an increase in lysosomal mass in aging cells and it is widely regarded as a marker for senescence [21]. NT2/D1 cells grown without curcuminoids showed no SA-β-galactosidase staining (Fig. 3D), whereas cells incubated with 6–7 µM curcuminoids showed increasingly vigorous staining after 4 and 12 days (Fig. 3E, F). Altogether, the irreversible withdrawal from the cell cycle, the morphological changes of the cells, and the detection of SA-β-galactosidase at pH 6 suggested that exposure to curcuminoids at 6–7 µM concentrations induced terminal cessation of cell division and cellular senescence. Although the images in Fig. 3 illustrate the effects of SOLID-solubilized curcuminoids, the results were indistinguishable with DMSO-solubilized curcuminoids.

A similarly prominent senescent phenotype could also be readily observed with CCF-STTG1 cells, albeit at the higher 20–22 µM concentration of SOLID-solubilized curcuminoids (not shown). In contrast, HeLa cells displayed a less pronounced senescent phenotype that occurred within a narrow 18–19 µM curcuminoid concentration range (Table 1). It is thus conceivable that curcuminoids may not induce senescence in all cell lines, or that the required concentration range to achieve this response is exceedingly narrow.

### Curcuminoids Induce Cell Death by Apoptosis

**Figure 4:**
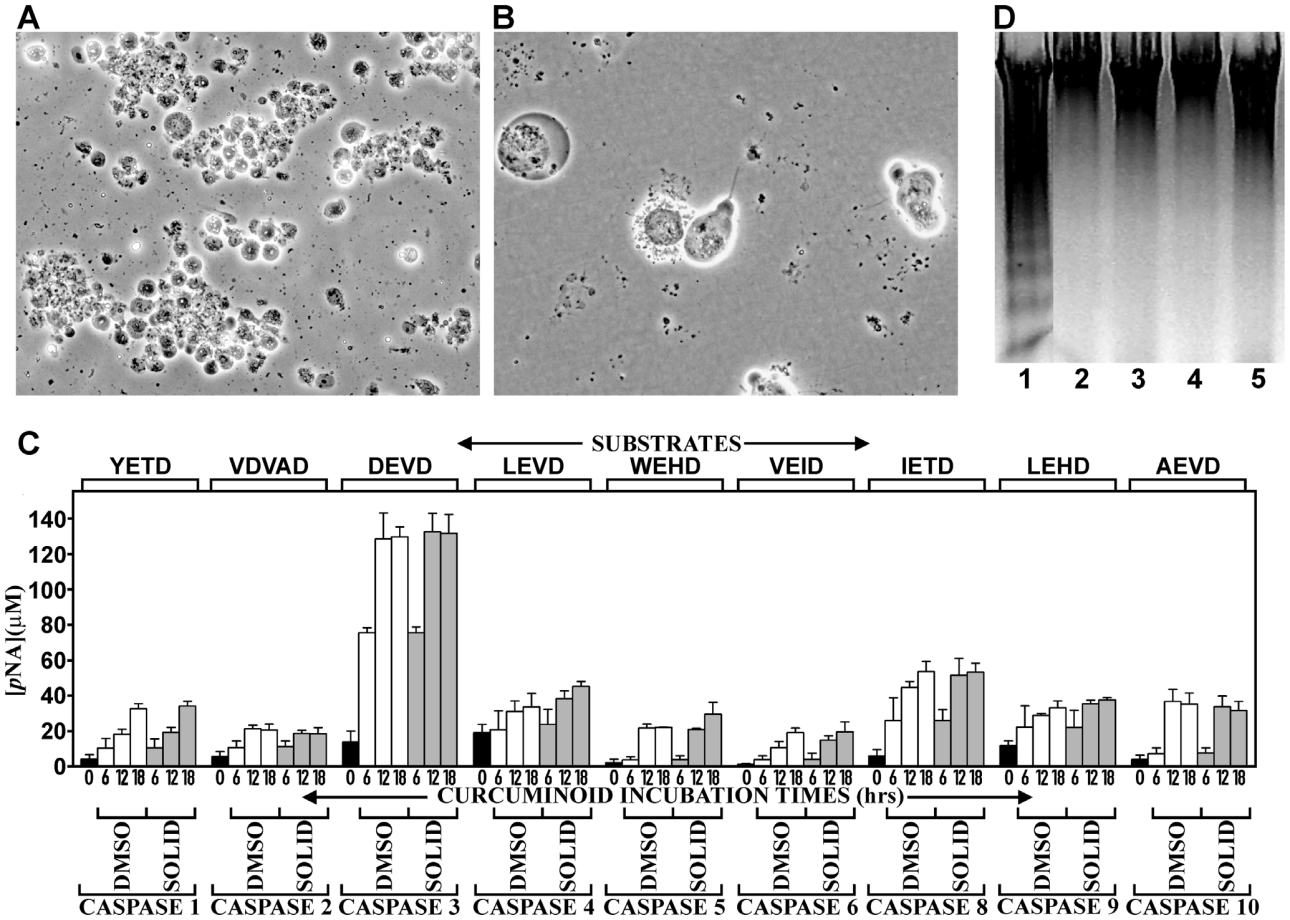
Induction of apoptosis by curcuminoids. (A) Phase contrast micrograph of NT2/D1 cells incubated with 47 µM media concentration of DMSO- solubilized curcuminoids for 18 h. Extensive cell death is indicated by rounding and detachment of cells. (B) At higher magnification, morphological changes consistent with apoptosis are observed. (C) Caspase activation by curcuminoids (46–49 µM) incubated for 0 (black bars), 6, 12, or 18 h either with SOLID- (gray bars) or DMSO-solubilized (white bars) curcuminoids. The activities reported for caspase 3 are to be considered a combination of the activities for caspases 3 and 7, since these share the same substrate and inhibitor specificity. (D) Detection of oligosomal DNA fragmentation by electrophoresis in 1% agarose and ethidium bromide staining. Lane 1: NT2/D1 cells after 6 h of incubation with 5 µM camptothecin, lane 2: Cells incubated without additives, lanes 3–5 cells incubated with 49 µM curcuminoids (DMSO-solubilized) for 6 h (lane 3), 12 h (lane 4), and 18 h (lane 5).

Since curcuminoid concentrations above 8–10 µM resulted in complete cell death within four days of incubation, the mechanism by which this occurred was further examined. For this purpose, NT2/D1 cells were incubated for shorter periods with maximal 46–49 µM curcuminoid concentrations. At these high concentrations, pervasive cell death was observed within 24 h of incubation, which facilitated the assays for apoptotic mechanisms. The structural changes in the cells included membrane blebbing, dissolution of the nuclear membrane, and cell fragmentation, which are all manifestations of apoptosis (Fig. 4A, B). Indeed, these structural responses coincided with a generalized activation of caspases. Maximum activation was uniformly observed after 12–18 hours of incubation (Fig. 4 C). The most prominent activation occurred with caspases 3/7. The additional activation of caspase 8 and to a lesser extent caspase 9, indicated a mixture of concurrent or sequential extrinsic and intrinsic pathways. However, the activation of other caspases suggested a more universal activation mechanism at these curcuminoid concentrations. Conversely, apoptosis-associated chromosomal DNA fragmentation could not be demonstrated with either curcuminoid preparation, whereas DNA fragmentation was observed in comparable apoptotic cultures incubated with 5 µM camptothecin for 6 h (Fig. 4D).

Overall, these results show that at low curcuminoid concentrations (<5 µM) the rate of cell division diminished and at higher concentrations (>8 µM) cell death by apoptosis was induced in a dose dependent manner. However, even at curcuminoid concentrations as high as 46–49 µM, it took about 12–18 h before morphological changes became apparent and 6 h before caspase activation was observed. At intermediate (6–7 µM) curcuminoid concentrations, cell division initially ceased and cellular morphology began to change. This early step could represent a form of cellular differentiation, although a specific cell type has not yet been determined. Upon longer exposure, cells irreversibly withdraw from the cell cycle and adopt a morphology consistent with senescence. In all instances, similar results were observed with curcuminoid preparations containing predominantly CUR (DMSO-solubilized) or BDMC (SOLID-solubilized).

### Curcuminoids Incubated with NT2/D1 Cells are Rapidly Depleted from the Medium

The dose-response effects observed with NT2/D1 cells were obtained with initial curcuminoid concentrations in media that were replenished every 24 h. The fate of the curcuminoids in cells and media during the period between media replacements was further examined, as it would depend on factors such as cellular binding, metabolism, and chemical decomposition. For this purpose, cells were incubated with media containing a 27 µM starting concentration of curcuminoids (DMSO+SOLID) for 5 h.

**Figure 5:**
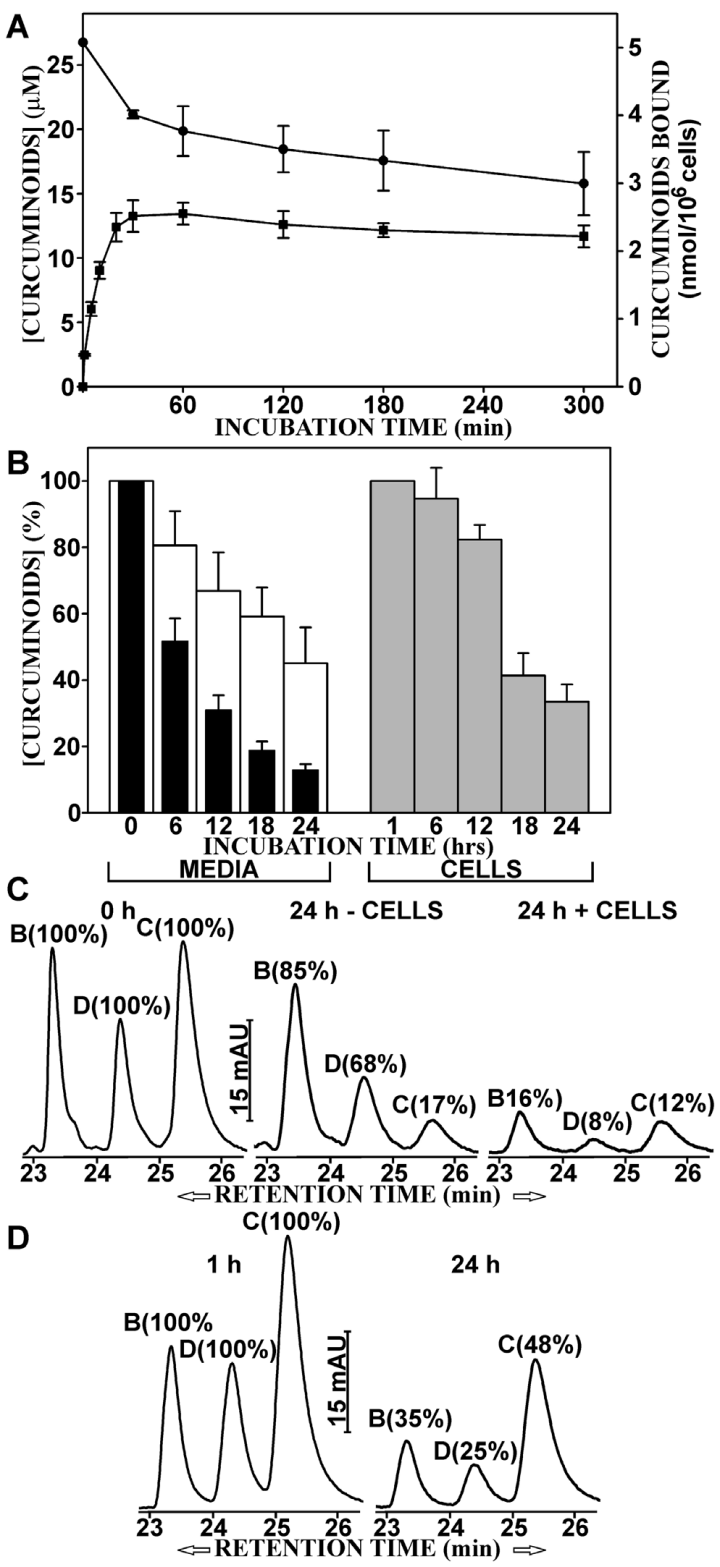
Curcuminoids bound to NT2/D1 cells and their concentration in media as a function of incubation time. (A) Cells were incubated with media containing 27 µM starting concentrations (0 min) of curcuminoids (SOLID+DMSO). The amount of curcuminoids bound to cells (▪, right axis) and their concentration in media (•, left axis) during 5 h of incubation. (B) Cells incubated for 24 h with media containing a 27 µM starting concentration (100%) of curcuminoids (SOLID+DMSO). Left panel: The relative concentrations (%) of curcuminoids in the media after 0, 6, 12, 18, and 24 h of incubation either in the presence (black bars) or absence (white bars) of cells. Right panel: The amounts of curcuminoids bound to cells after 1, 6, 12, 18, and 24 h of incubation relative to the amount of curcuminoids bound to cells after 1 h of incubation (100%). (C) Representative chromatograms showing the elution profiles of curcuminoids in media before incubation (0 h, left panel) and after 24 h of incubation without cells (center panel) or with cells (right panel). The integrated and adjusted (ε) values under each individual peak are expressed relative to the respective starting concentrations (100%). (D) Curcuminoid binding to cells after 1 h (left panel) and 24 h (right panel). Curcuminoids bound after 24 h of incubation are expressed relative to the amount bound after 1 h (100%).

Within 5 h of incubation, the curcuminoid concentration in the media had decreased to about 59% of the original value. About half of that decrease occurred during the first 0.5 h of incubation, which was followed by a more gradual decline for the remaining period. The initial media depletion of curcuminoids coincided with the establishment of maximum cellular binding, which was attained after about 0.5 h. Thereafter, the levels of cellular-bound curcuminoids effectively remained unchanged for the remaining 5 h incubation period (Fig. 5A). These cellular binding data are similar to those observed in other studies [22], [23].

Upon longer incubation, media curcuminoid concentrations continued to diminish from about 50% of the original value after 6 h to 12% after 24 h (Fig. 5B). In media incubated under the same conditions without cells, curcuminoid concentrations also decreased, albeit at a slower and essentially linear rate. Although initially stable, cellular binding of curcuminoids significantly decreased between 6 and 24 h of incubation (Fig. 5B).

Comparing chromatograms of curcuminoids extracted from media incubated with or without cells allowed for a more differentiated evaluation of the results. In media incubated without cells, the concentration of CUR had decreased to about 17% of the starting concentration within 24 h, whereas BDMC and, to a lesser extent, DMC were more stable. In contrast, in media incubated with cells, all three curcuminoids had decreased to values of less than 16% (Fig. 5C). By comparison, the binding of CUR to cells had only declined to 48% of the original value (1 h), whereas the binding of BDMC and DMC had decreased to 35% and 25%, respectively (Fig. 5D).

These results show that both media concentrations and cellular binding of curcuminoids significantly decreased during the 24 h incubation period. Some of this concentration decline in the media can be accounted for by chemical decomposition [13], [24], which is evident in media incubated without cells. Indeed, under these conditions most of the decrease in curcuminoid concentration can be attributed to the chemical instability of CUR. In contrast, BDMC and to a lesser extent DMC are remarkably stable. In the presence of cells, initial cellular binding within the first 0.5 h of incubation also contributes to the decline in media curcuminoid concentration. Indeed, the robust binding of CUR to cells after 24 h of incubation, despite low levels in the media, suggests that this binding occurred at an earlier stage and that CUR bound to cells is more chemically stable. Overall, the data suggest that the decline in CUR concentration in the media is primarily due to chemical decomposition and, to a lesser extent, early cellular binding and metabolism. In contrast, the removal of BDMC and DMC from the media and the subsequent lower levels of cellular binding implies robust cellular metabolism of these compounds, which would be considerably slower for CUR.

### Cellular-bound Curcuminoids are Primarily Associated with Membrane Fractions

**Figure 6:**
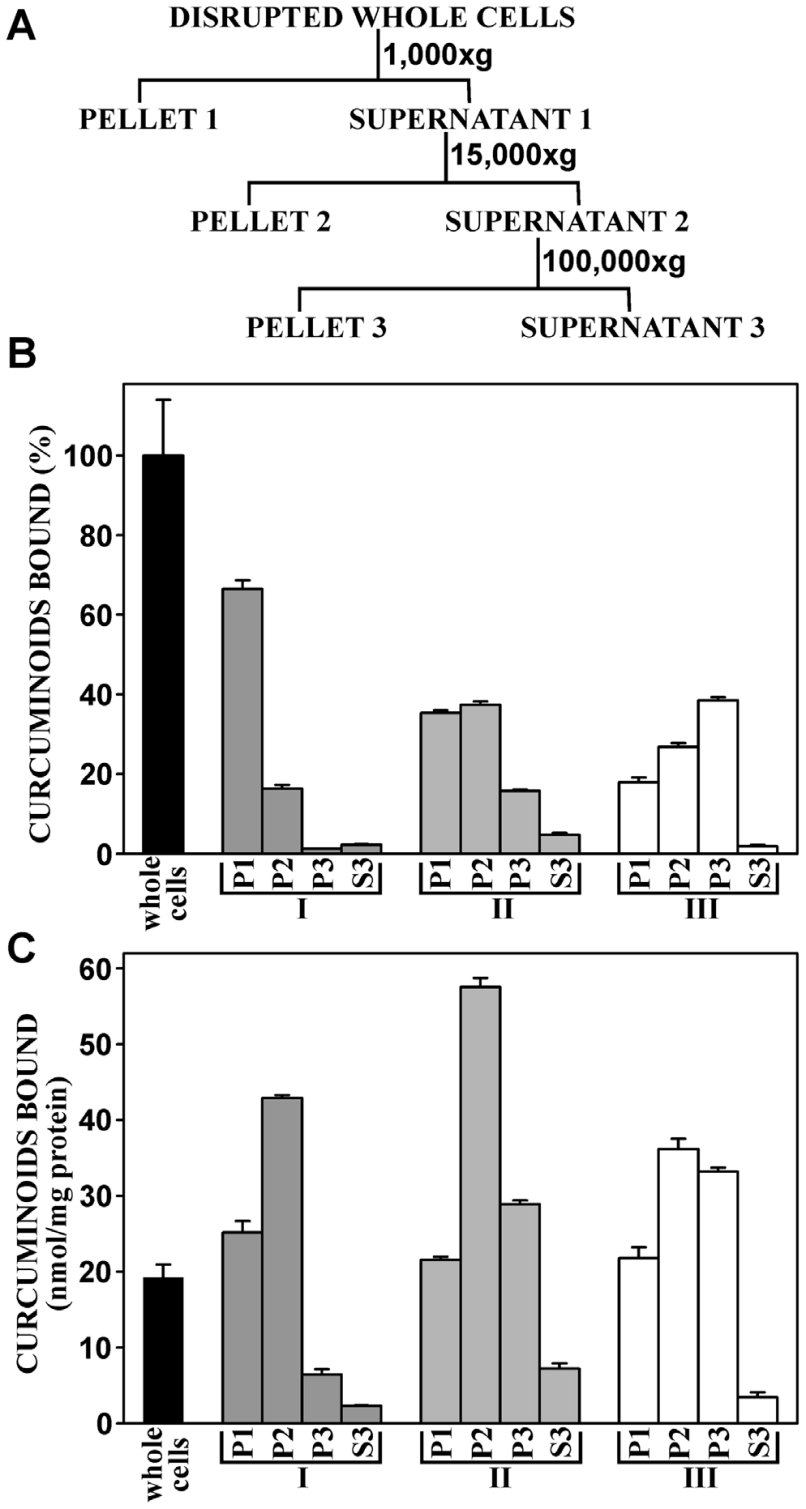
Subcellular fractionation of curcuminoids bound to NT2/D1 cells. (A) Schematic outline of fractionation procedure. (B) Relative distribution of total curcuminoids in centrifugal pellets 1–3 (P1–P3) and final supernatant (S3) as outlined in A. Cells were disrupted by either by homogenization (I), freeze-thawing (II) or sonication (III) in hypotonic medium. The amounts of curcuminoids in the respective fractions are expressed relative to the amount of curcuminoids bound to whole cells (100%). (C) Same as in B except that curcuminoids were normalized to the amount of protein in the respective fractions.

To elucidate the subcellular target for cellular binding, NT2/D1 cells were incubated with curcuminoids at a concentration of 47 µM (SOLID+DMSO) for 1 h, at which time maximum cellular binding had been firmly established. To produce different degrees of cellular fragmentation, cellular disruption was achieved either by gentle homogenization in hypotonic buffer, three cycles of freeze-thawing combined with intermittent vortexing with glass beads, or by sonication. The cell-free preparations were then subjected to three successive centrifugations (Fig. 6A). The first low-speed centrifugation (1,000×g) separated unbroken cells, large membrane fractions (cellular ghosts) and nuclei (pellet P1). The supernatant (S1) of that step was centrifuged at higher speed (15,000×g) to separate intermediate sized membrane fractions and organelles (pellet P2). The supernatant (S2) of that step was further centrifuged at 100,000×g to pellet residual membrane vesicles not separated at lower speeds (P3). The remaining supernatant (S3) consisted largely of cytoplasmic material.

When cells were gently homogenized in hypotonic medium (Fig. 6B, C, I), microscopic examination revealed predominantly large membrane fractions (cellular ghosts) with free or trapped nuclei, and few unbroken cells. Based on the total distribution of curcuminoids, most of the binding activity occurred in pellet P1, indicating that curcuminoids were bound to unbroken cells or large membrane fractions. Some curcuminoid binding was also present in pellet P2, and very little binding in pellet P3 and cytoplasmic supernatant S3 (Fig. 6B). When the curcuminoid binding activity was normalized to protein content in the various fractions, the highest binding activity was observed in pellet P2 (Fig. 6C). In both instances, the cytoplasmic supernatant had exceedingly low curcuminoid content (Fig. 6B, C).

Cells that were disrupted by freeze-thawing (Fig. 6B, C, II), produced largely intermediate sized membrane fragments together with free nuclei and smaller fragments appearing as cell debris. As a result, curcuminoid binding shifted away from pellet P1 to the higher speed pellets P2 and P3 (Fig. 6B, C). This trend also extended to fractions normalized to protein content (Fig. 6C).

After sonication (Fig. 6, III), cell disruption was exhaustive with few intermediate sized membrane fractions and larger amounts of cell debris. Although many of the nuclei had also been disrupted, no free nucleic acids were evident in the buffer. Under these harsher conditions of cell disruption, curcuminoid binding was transferred further toward the high speed pellet P3 at the expense of pellets P1 and P2. Again, little or no curcuminoid was detected in cytoplasmic fraction S3 (Fig. 6B, C).

Differential centrifugation by itself is inherently unreliable in the purification of subcellular particles. This is due to excessive cross-contamination with membrane particles of variable sizes with specific organelles of interest. For an exhaustive characterization, further purification by equilibrium centrifugation in sucrose gradients would be essential. However, such centrifugations are usually quite long (>16 h) and in the case of curcuminoid binding, the stability of the binding complex would be a serious concern. Nonetheless, for a preliminary assignment of the curcuminoid binding activity, differential centrifugation was here applied to cells that had been subjected to increasingly harsher disruption procedures. The results clearly demonstrate that the overwhelming curcuminoid binding activity is located to membrane fractions. The precise membrane components associated with this binding remain to be determined. However, further results discussed below suggest that the cell membrane is the primary binding target. Although a significant proportion of binding activity remained in the nuclear pellet (1,000×g) even after sonication, it is unlikely that nuclear binding is a substantial contributor. In particular, since nuclei purified by detergent methods show little or no binding activity when incubated directly with curcuminoids (not shown).

### The Dose-dependent Binding of Curcuminoids to NT2/D1 Cells is Saturable and Competed by Free Serum

**Figure 7:**
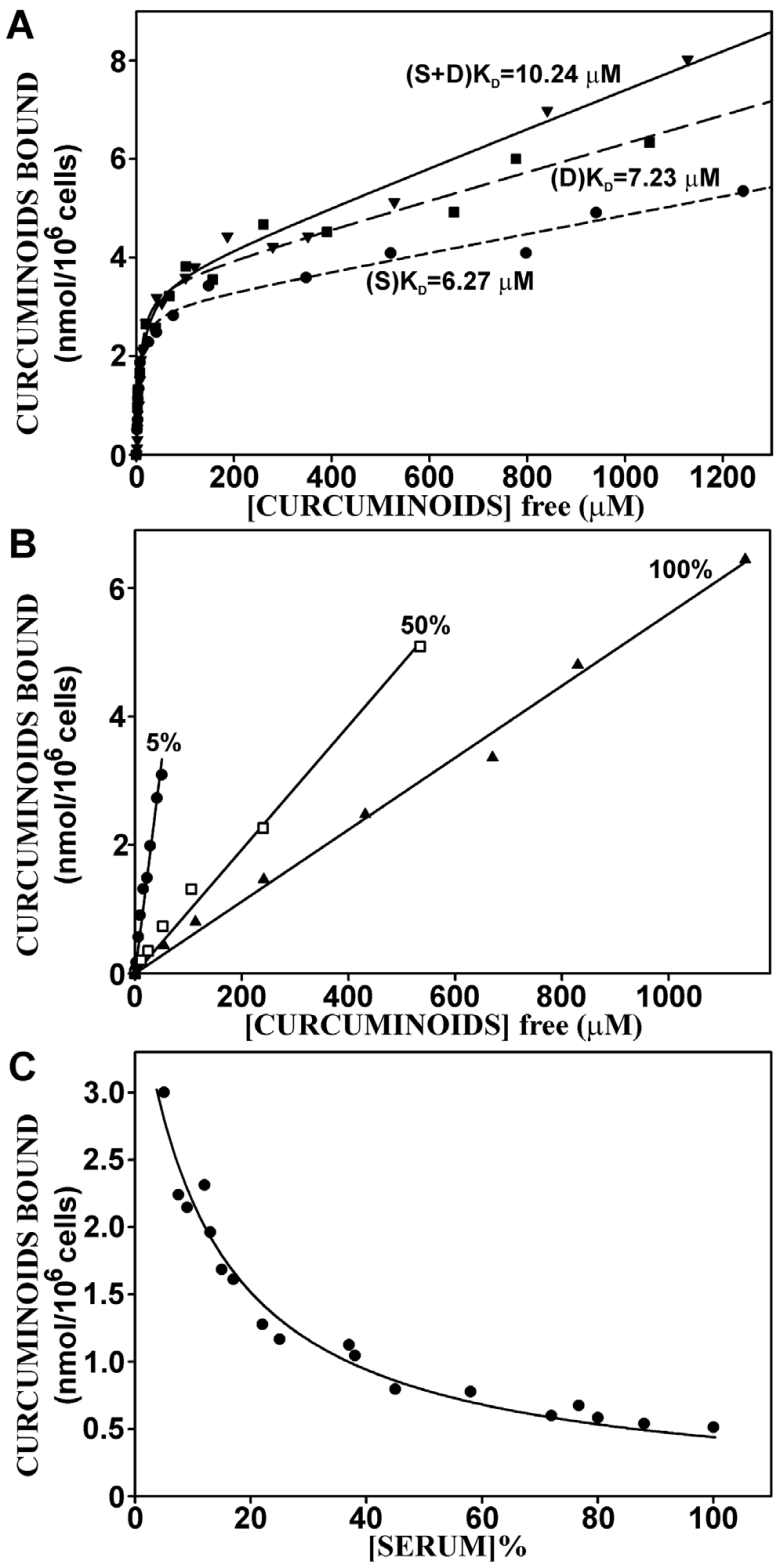
The dose-dependent binding of curcuminoids to NT2/D1 cells. (A) Binding dose curves with variable serum and curcuminoid concentrations in media containing FCS with saturated SOLID(S)-[•, short dash] and DMSO(D)-solubilized [▪, long dash] curcuminoids, and a 1∶1 mixture of the two (S+D) [▾, solid line]. The data points represent values from three independent experiments normalized to cell number. The following additional statistical parameters were calculated: R^2^-values: 0.99 (S), 0.98 (D), and 0.99 (S+D); K_D_s: 6.27±1.03 µM (S), 7.23±1.54 µM (D), 10.24±1.36 µM (S+D); and the slope m (see equation in text) indicating degree of non-specific binding: 1.9×10^−3^ (S), 2.9×10^−3^ (D), and 3.9×10^−3^ (S+D). (B) Binding dose curves with variable curcuminoid concentrations (S+D) at three constant total serum concentrations (5%, 50%, and 100%). (C) Binding dose curve with constant 47 µM curcuminoid concentration (S+D) and variable serum concentration.



The overall equilibrium dissociation constants (K_D_) sites curcuminoids bound to NT2/D1 cells was determined by incubating increasing amounts of saturated serum-solubilized curcuminoids with NT2/D1 cells for 1 h. The amount of cellular-bound and the concentration of unbound curcuminoids were determined and the resulting data were fitted to binding curves (Fig. 7A). The simplified premise here is that soluble curcuminoids (C_SOL_) in the media bind to cellular sites that will here for operational purposes be referred to as receptors (R). Such soluble curcuminoids would then form the complex C-R with the cellular binding sites as described by the equilibrium:

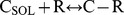



Since the total number of receptors available for specific binding (R_T_) is proportional to the number of cells and the number of unbound receptors [R] = [R_T_]-[C-R], the dissociation constant is described by the equation:
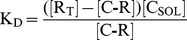
Rearranged and with the factor m[C_SOL_] added to account for non-specific binding:



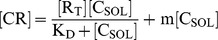
The thus obtained apparent overall K_D_s for total curcuminoid binding to NT2/D1 cells were similar for SOLID-solubilized (K_D_ = 6.27 µM) and DMSO-solubilized (K_D_ = 7.23 µM) curcuminoids. However, when the SOLID- and DMSO-solubilized curcuminoids were mixed, the apparent K_D_ increased to 10.24 µM. The degree of non-specific binding, which was reflected by the slope (m) of the linear segment of the curve (m[C_SOL_]), depended on the method of curcuminoid-solubilization and increased in the sequence SOLID<DMSO<SOLID+DMSO (Fig. 7A).

Curcuminoid-binding to HeLa and astrocytoma CCF-STTG1 cells occurred with a 3- to 4-fold lower affinity than to NT2/D1 cells. The K_D_-values ranged from 17 µM (HeLa) and 25.6 µM (CCF-STTG1) for SOLID-solubilized to 22.2 µM (HeLa) and 29.9 µM (CCF-STTG1) for DMSO-solubilized curcuminoids. In contrast, the maximum specific binding of curcuminoids to each cell line (B_MAX_) was less variable (Table 1).

When binding of individual curcuminoids to NT2/D1 cells was plotted against total free curcuminoids, the apparent K_D_ values for individual curcuminoids increased in the order BDMC (2.81–5.49 µM)<DMC (6.16–8.70 µM)<CUR (8.68–12.05 µM). Furthermore non-specific binding was considerably more pronounced with CUR than with BDMC or DMC. This resulted in an increase in relative binding of CUR over BDMC and DMC, which was particularly apparent at higher soluble curcuminoid concentrations (compare Fig. 1). It should also be noted that pure synthetic curcumin and analytical grade curcumin (>90% CUR) resulted in higher apparent K_D_ values of 19.05 and 18.13 µM, respectively (not shown).

Apparent K_D_ values determined with curcuminoids solubilized in 10% albumin were as follows: SOLID: 7.29 µM, DMSO: 8.35 µM, and SOLID+DMSO: 19.85 µM. Non-specific binding to cells incubated with albumin-solubilized curcuminoids was considerably lower or non-existent compared to that observed with serum-solubilized curcuminoids (not shown).

The incubation conditions applied here denote increased curcuminoid concentrations obtained with curcuminoid-saturated serum. As a result, increasing curcuminoid concentrations were also linked to higher serum concentrations. These conditions were different from those employed for experiments described for Fig. 2, where the total serum concentration was held constant at 5% and the concentration of curcuminoid-saturated serum was varied.

The influence of serum on curcuminoid-binding to cells was therefore explored at constant serum and variable curcuminoid concentrations. NT2/D1 cells were incubated with media containing increasing concentrations of curcuminoids (SOLID+DMSO) at constant total serum concentrations of 5%, 50%, or 100%. Within each of the three total serum concentrations examined, the amount of cellular bound curcuminoids increased linearly with the concentration of unbound curcuminoids. Comparable results were obtained with SOLID- and DMSO-solubilized curcuminoids (not shown). This indicates that cellular binding was not saturable within the parameters limited by the maximum solubility of curcuminoids in serum. Furthermore, the cellular binding of curcuminoids decreased with increasing serum concentration, which is evident from a decline in the slope of the binding curves as the serum concentrations increased (Fig. 7B). These results were different from those obtained in Fig. 7A, where binding saturation was observed when cells were incubated with increasing amounts of curcuminoid-saturated serum. This suggests that the additional curcuminoid-free serum acts as a *de facto* competitor for cellular curcuminoid binding.

This notion was further confirmed by examining the effect of increasing serum concentrations on cellular binding at a constant 47 µM (SOLID+DMSO) curcuminoid concentration in a total initial 5% serum concentration (Fig. 7C). The data points were best described by a hyperbolic decline function (R^2^ = 0.97) described by the equation:
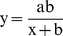
This equation is consistent with the notion that free curcuminoids in solution primarily exist in a complex with serum components (C-S) that bind to cellular receptors (R) to form a curcuminoid-receptor complex (C-R) illustrated by the equilibrium:



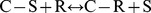
This yields the dissociation constant:



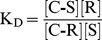
With [R] = [R_T_]-[C-R] and [S] = [S_T_]-[C-S], and rearranged:



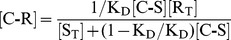
When K_D_<<1, the term (1-K_D_/K_D_)→1/K_D_, and with [C-S] and [R_T_] presumed to be constant, the equation assumes the form of the original function used for curve fitting. Therefore, as the free serum concentration increases, the curcuminoid-binding equilibrium is shifted from the cellular receptors to the serum complex. Consequently, the availability of free serum in essence acts as a competitor for curcuminoid-binding to cells.

### Curcuminoids are Differentially Metabolized by NT2/D1 Cells

The fate of cellular-bound curcuminoids was further examined by first allowing binding equilibrium to be established followed by the removal of curcuminoids from the media. NT2/D1 cells were therefore initially incubated with media containing 47 µM curcuminoids (SOLID+DMSO) for 1 h. The cells were then washed and incubated without curcuminoids for another 2 h either with DMEM media alone or supplemented with 5% FCS. The fate of cellular-bound curcuminoids was also examined under subsequent cell-free conditions. In this case, cells were instead incubated with 10 mM hepes, pH 6.8 at 37°C. This hypotonic condition resulted in rapid swelling and rupture of cells, leaving a suspension of membranes and cytoplasmic content.

**Figure 8:**
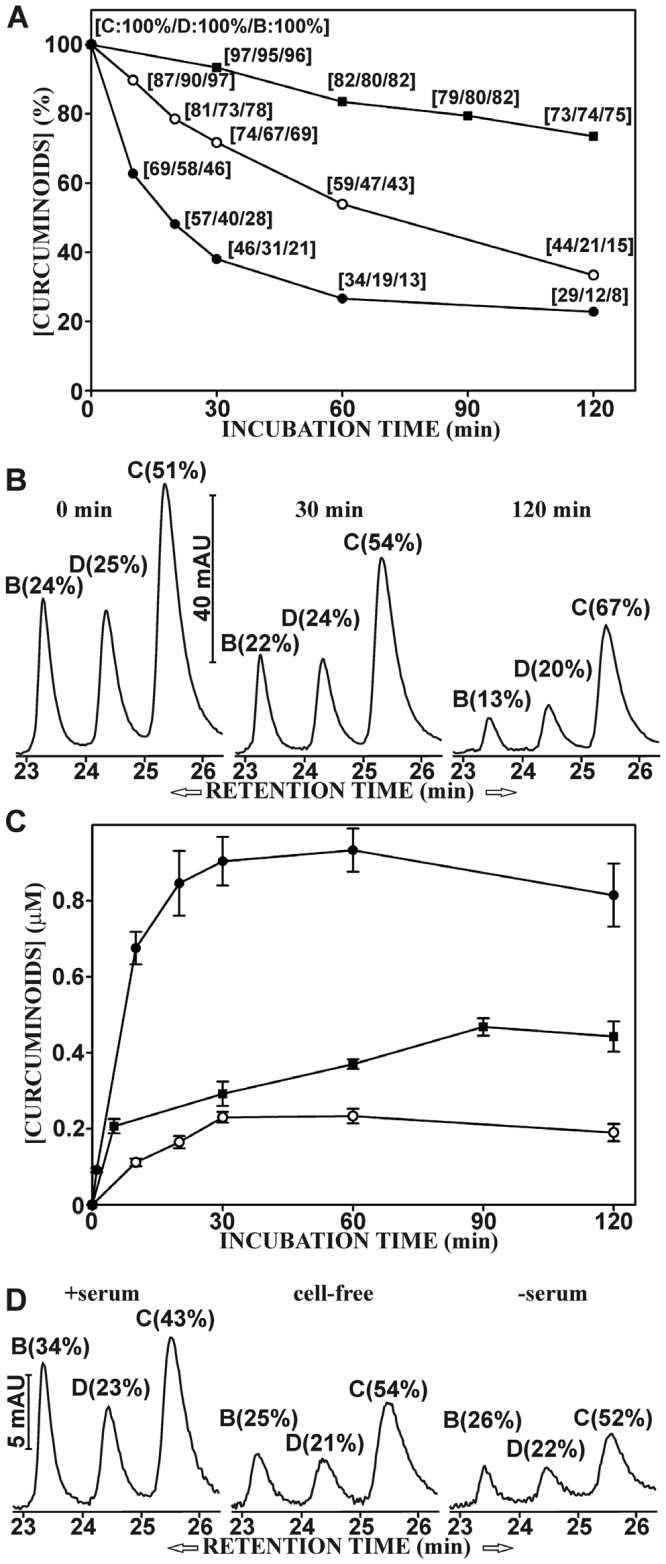
The dissociation of curcuminoids from NT2/D1 cells. (A). Cells were incubated with media containing 47 µM curcuminoids (SOLID+DMSO) for 1 h. The media were then removed and the cells washed. Cells were then incubated either with 2 ml of DMEM alone (○) or DMEM +5% FCS (•). Alternatively, cells were hypotonically lysed and incubated with 2 ml of 10 mM hepes, pH 6.8 (▪). The amount of curcuminoids bound to cells was then measured during a 2 h period. The numbers in brackets represent amounts of CUR (C), DMC (D) and BDMC (B) relative to their respective starting concentrations (100%). The values represent the average of three independent experiments with standard deviations ranging from 3–8% (error bars omitted). (B) Elution profiles of curcuminoids bound to cells at the start of incubation (0 min) and after 30 and 120 min. The numbers in parentheses indicate relative molar amounts of curcuminoids bound. (C) The concentrations of curcuminoids released into the media after incubation as described in A. (D) Elution profiles of curcuminoids in media after 30 min of incubation in DMEM alone (-serum), in DMEM with 5% FCS (+serum) and in 10 mM hepes, pH 6.8 (cell-free).

During the 2 h incubation period, the amount of cellular-bound curcuminoids declined rapidly. In cells incubated with DMEM media without serum, the cellular-bound curcuminoids declined in an essentially linear manner to a level of about 32% of the original value. In serum-containing media, the drop in the level of cellular-bound curcuminoids was more pronounced during the first 0.5 hour of incubation and the rate of decline slowed significantly thereafter to reach a final level of about 20% of the original value. Under cell-free conditions in hypotonic buffer, the decline in the amount of membrane-bound curcuminoids was slower and also essentially linear with time. At the end of the 2 h incubation time, over 70% of the curcuminoids remained bound to the membrane fraction (Fig. 8A).

Although the levels of all bound curcuminoids decreased during incubation with DMEM media, the relative decline was more rapid for BDMC and DMC than for CUR (Fig. 8A). This was also reflected in a time-dependent increase in the level CUR and a decrease in the levels of BDMC and DMC relative to the total amount of bound curcuminoids (Fig. 8B). In contrast, the decline in the levels of individual curcuminoids proceeded at the same rate under cell-free conditions (Fig. 8A). These results suggest that curcuminoids are actively metabolized by intact cells and that the rate of metabolism is faster for BDMC and DMC than for CUR.

A parallel determination of the curcuminoid concentration in cell culture media showed a rapid increase within the first 0.5 h of incubation. The maximum curcuminoid concentration in serum-containing media was about five-fold higher than in serum-free medium and more than two-fold higher than in cell-free buffer (Fig. 8C). More importantly, the curcuminoid composition differed between serum-containing and serum-free media (Fig. 8D). In serum-containing media, the curcuminoid composition was similar to that of the original incubation medium (compare Fig. 1A, B, right panel), whereas in serum-free media the curcuminoid composition was similar to that of the cellular-bound curcuminoids (compare Fig. 1C, right panel).

These results confirm that the cellular binding of curcuminoids is at least partially reversible, which strongly suggests their presence on the cell membrane (Fig. 6). With serum-containing media, the original equilibrium is reestablished between cells and curcuminoid-binding components of serum. In contrast, in serum-free media the equilibrium is established between cellular-bound curcuminoids and the aqueous media, resulting in a media composition that is identical to that of curcuminoids bound to cells. The higher concentration of curcuminoids in serum-containing medium would then be due to their higher solubility in serum. The intermediate concentration in the cell-free supernatant may reflect either curcuminoid binding to cytoplasmic components following their release into the buffer or partial contamination with small cell membrane fragments that did not sediment at 18,000×g. The continued differential decrease in cellular-bound curcuminoids suggests active metabolism by intact cells, a notion that is supported by the lack of similar decreases in cell-free incubations (Fig. 8A).

### Detection of Metabolic Products of Curcuminoids in Media

**Figure 9:**
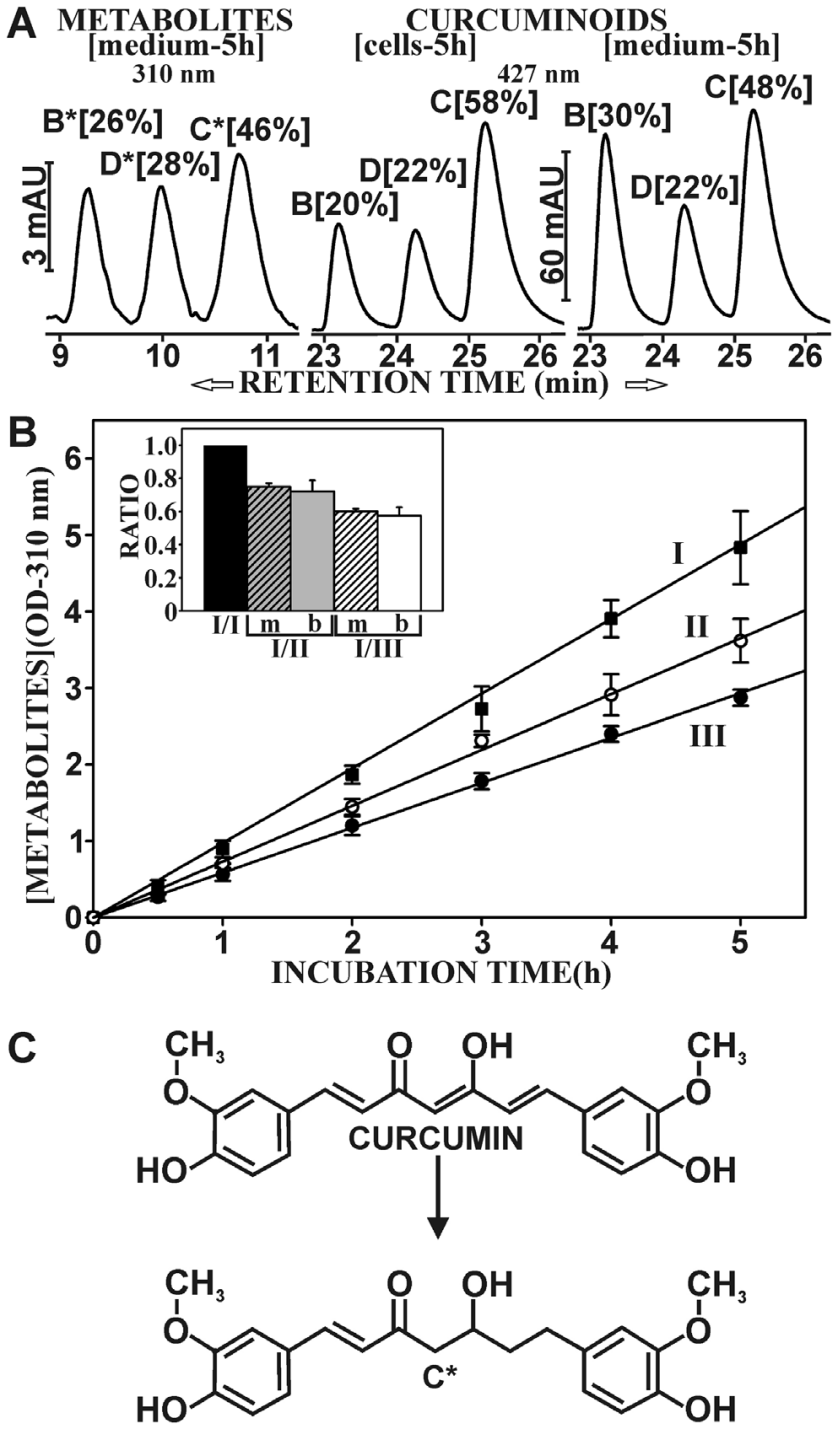
Metabolites of curcuminoids detected in incubation media. (A) NT2/D1 cells were incubated with media containing 47 µM curcuminoids (SOLID+DMSO) for 5 h. Left panel: The elution profile at 310 nm wavelength of metabolites derived from BDMC (B*), DMC (D*), and CUR (C*) in the media. Center and right panels: The elution profiles at 427 nm wavelength of curcuminoids extracted from cells and media, respectively. The relative areas under the curve of each peak are indicated in square brackets. (B) Time course of the generation of curcuminoid metabolites (OD at 310 nm wavelength) in the media under three incubation conditions: Incubation of cells with 47 µM curcuminoids in 5% FCS (I, ▪), with 24 µM curcuminoids in 2.5% FCS (II, ○), or with 24 µM curcuminoids in 5% FCS (III, •). Insert: The value of the slope (m) of curve II (I/II, hatched bar) and III (I/III, hatched bar) relative to the value of the slope of curve I (I/I, black bar). The resulting ratios were similarly compared to the ratios of curcuminoids bound to cells (b, solid bars) after 5 h under the same respective incubation conditions (C) Putative structure of the metabolic product of CUR (C*) based on the evaluation of mass spectrometry and absorption properties.

Since no metabolic products could be detected by reversed phase chromatography at an absorption wavelength of 427 nm, the search for metabolic products in cells and incubation media was further extended to include wavelengths 280 nm and 310 nm. During incubation of NT2/D1 cells with media containing 47 µM curcuminoids (SOLID+DMSO), absorption peaks with a similar elution profile as the curcuminoid peaks at 427 nm were detected at an absorption wavelength of 310 nm (Fig. 9A). These compounds eluted faster, indicating higher polarity and they also absorbed at 280 nm wavelength (not shown). These properties suggested that the products were structurally related to curcuminoids and metabolically derived from the parent compounds. The elution profile of the metabolic products was more similar to the elution profile of curcuminoids bound to cells than that of the medium (Fig. 9A). However, the metabolic products of BDMC (B*) and DMC (D*) were relatively more prevalent in the medium (26% and 28%, respectively) after 5 h of incubation than their corresponding parental compounds (20% and 22%, respectively) bound to cells. This implies that BDMC and DMC were metabolized faster than would be suggested by their relative cellular binding, which is consistent with the observation that these compounds were in relative terms eliminated faster from cells and media than CUR (Figs. 5 and 8A).

The curcuminoid metabolites were exclusively found in the media and could not be detected in cells. Furthermore, these peaks were absent in media without cells and their accumulation in the media increased linearly in a time dependent manner over a 5 h incubation period (Fig. 9B). Cells were incubated either with 47 µM saturated curcuminoids (SOLID+DMSO) in a total media serum concentration of 5% (curve I), 24 µM saturated curcuminoids in a total media serum concentration of 2.5% (curve II), or 24 µM saturated curcuminoids diluted 1∶1 with regular serum for a total media serum concentration of 5% (curve III) (Fig 9B). In all three cases, there was a linear accumulation of metabolic products as a function of time (R^2^>0.99), although the rate of accumulation varied as reflected by the different slopes of the three curves. The three incubation conditions also resulted in differences in the amount of curcuminoids bound to cells. This was either due to concentration-dependent (curves I and II) or serum-dilution effects (curve III). Indeed, there is excellent agreement between the accumulation of metabolic product in the media and the amount of curcuminoids bound to cells after a 5 h incubation period (Fig. 9B, insert). Therefore, the metabolic conversion was proportional to the amount of curcuminoids bound to cells rather than their concentrations in the media.

**Figure 10:**
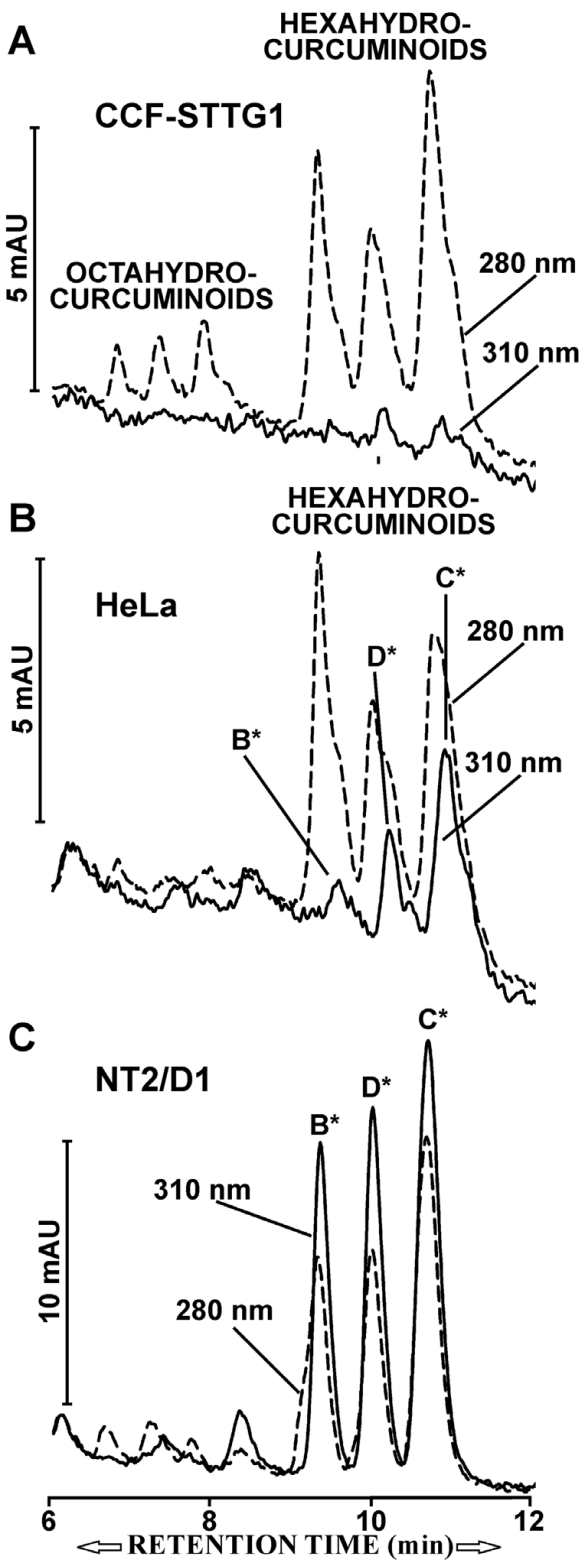
Metabolites of curcuminoids in different cell lines. (A) CCF-STTG1 cells were incubated with media containing 47 µM curcuminoids (SOLID+DMSO) for 24 h. During reversed phase chromatography of media samples, the elution profiles were monitored at wavelengths 280 nm (hatched lines), and 310 nm (solid lines). Prominent absorption peaks representing hexa- and octahydrocurcuminoids were detected at 280 nm wavelength. (B) Primarily hexahydrocurcuminoids were produced with similarly incubated HeLa cells, in addition to smaller amounts of curcuminoid reduction products C*, D*, and B*. (C). In contrast, NT2/D1 cells generated only reduction products C*, D*, and B*.

The faster elution times and the loss of absorption at longer wavelengths compared to the parent compounds suggested reductive modifications within the conjugated keto-enol linker region of the curcuminoids. For structural assessment, the peak corresponding to the metabolic product of CUR was collected and further analyzed by mass spectrometry. This generated a compound with a molecular mass of 371, which was identical to that of tetrahydrocurcumin. However, a direct comparison with tetrahydrocurcumin revealed that the mass spectra of the two compounds were different although they shared some overlapping features. Furthermore, the spectral absorption of the two compounds differed. While tetrahydrocurcumin only absorbed at 280 nm, the curcuminoid metabolic products showed a higher absorption at 310 nm wavelength. More importantly, the elution peaks of the two compounds did not overlap. This indicated that the metabolic product identified here was different from tetrahydrocurcumin. It should here be noted that the elution peaks produced by hexahydrocurcuminoids, overlap extensively with the alternative metabolic products described here (Fig. 10). However, the absorption maxima do not align perfectly and hexahydrocurcuminoids do not absorb at 310 nm wavelengths. Based on the observations from mass spectrometry and the absorption characteristics of the compounds, the metabolic product of curcumin (C*) was tentatively assigned the structure 1,7-bis(4-hydroxy-3-methoxyphenyl)-5-hydroxy-1-heptene-3-one (Fig. 9C). The remaining carbon-carbon double bond within the aliphatic linker thus forms a more extended conjugated double bond system with the remaining keto-group than is the case in tetrahydrocurcumin, and the compound is therefore expected to absorb at longer wavelengths. However, the ultimate structure assignment would need to be confirmed by NMR.

The presence of metabolites in the incubation media was also assessed in CCF-STTG1 and HeLa cell cultures. After 24 h of incubation, the primary metabolites generated by CCF-STTG1 cells were hexahydrocurcuminoids together with smaller amounts of octahydrocurcuminoids (Fig. 10A). In contrast, HeLa cells generated no octahydrocurcuminoids (Fig. 10B). However, in addition to hexahydrocurcuminoids, HeLa cells also produced some of the same C*, D*, and B* metabolic products observed with NT2/D1 cells (Fig. 10C). Although HeLa and CCF-STTG1 cells produced primarily hexahydrocurcuminoids after exposure to curcuminoids for 24 h, the C*, D*, and B* metabolic products featured more strongly during the early stages (<5≤h) of incubation (not shown). These results indicate that cell lines differentially metabolize curcuminoids and the metabolic products may reflect the tissue of origin of the cell line.

### The Biological Effects of Curcuminoids can be Elicited by Short-term Exposure to High Concentrations of Curcuminoids

The described data (Figs. 2, 3, 4) show that continuous exposure of NT2/D1 cells to curcuminoids caused distinct biological responses in a dose-dependent manner. In those experiments, cells were exposed to varying initial concentrations of curcuminoids for a period of 24 h before being replenished with fresh medium. However, subsequent experiments on cellular binding and media depletion suggested that the cells actively metabolized curcuminoids, resulting in a decline in cellular-bound curcuminoids during the 24 h time period. There was also limited chemical decomposition of curcuminoids, independent of the presence of cells. As a result, the initial starting curcuminoid concentrations in the media were transient and significantly decreased during the 24 h incubation period. The experimental conditions therefore more accurately reflected exposure to successive starting concentrations of curcuminoids that were restored every 24 h. Consequently, it might be expected that different results would be obtained if the curcuminoid-containing media were replenished more often or even continuously.

**Figure 11:**
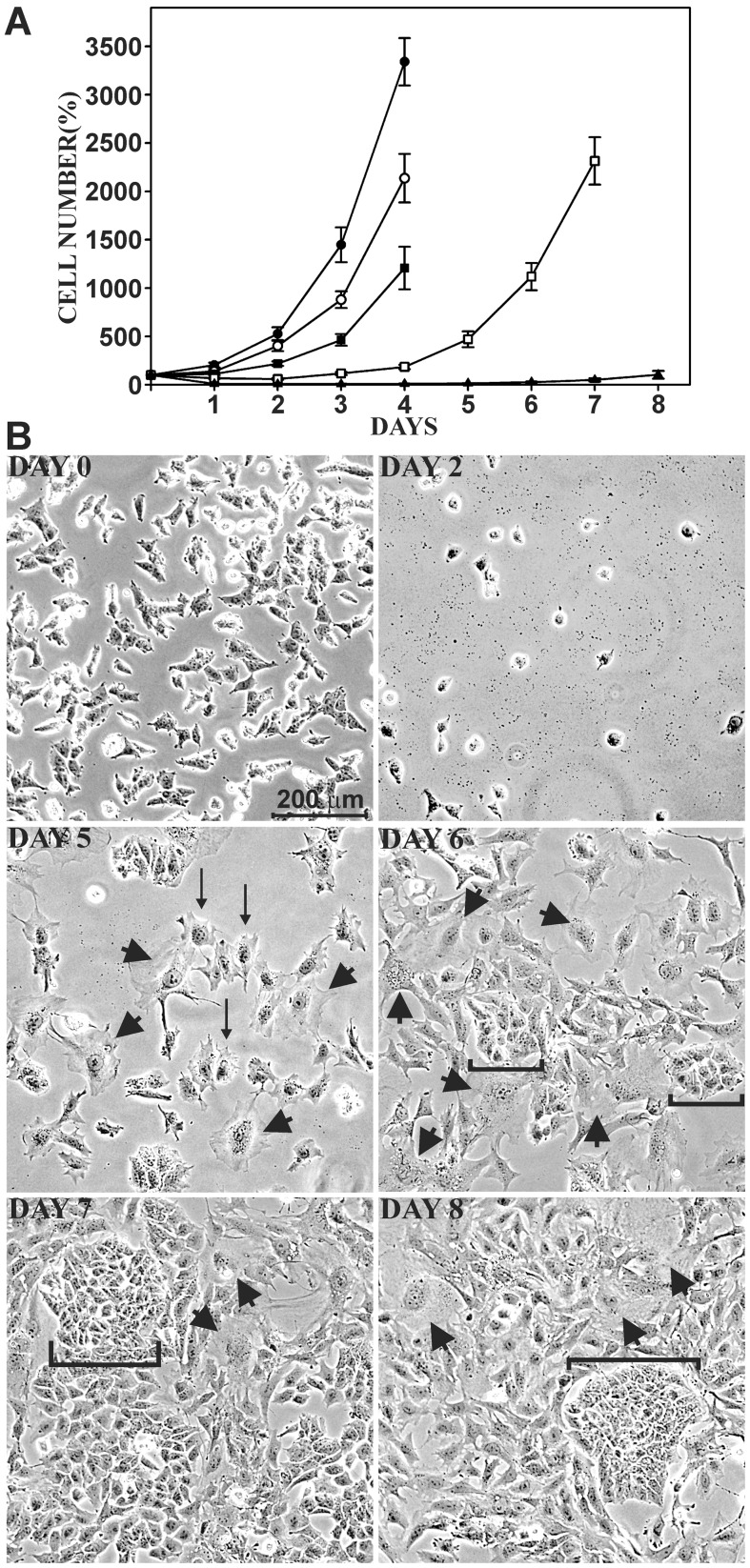
Effect of incubating NT2/D1 cells for short time periods with high concentrations of curcuminoids. (A) Cells were either incubated with standard medium (•) or with medium containing 47 µM curcuminoids (SOLID+DMSO) for 0.5 h (○), 1 h (▪), 2 h (□), or 4 h (▴). The media were then removed, cells washed, and replenished with standard medium. Cells were photographed and counted daily. (B) Phase contrast micrographs of NT2/D1 cells before (day 0) and 2, 5, 6, 7, and 8 days after incubation with 47 µM curcuminoids for 4 h. Arrows point to larger dividing cells and arrowheads to cells reminiscent of a senescent phenotype. Brackets delineate groups of cells that have reverted to the original phenotype (compare Fig. 3).

Alternatively, similar responses might be elicited by transiently exposing cells to higher curcuminoid concentrations. To examine this possibility, cells were incubated with 47 µM curcuminoids (SOLID+DMSO) for 0.5 h, 1 h, 2 h, and 4 h. Thereafter, cells were washed with PBS and incubated with curcuminoid-free standard medium. Cells were then monitored for 4–8 days to detect changes in the rate of cell division and evidence for morphological changes or senescence. When cells were exposed to 47 µM curcuminoids for 0.5 h and 1 h, cell proliferation initially decreased for a period of 1–3 days after exposure. Thereafter, the rate of cell division resumed at normal levels, however with lower starting cell numbers than the untreated control. No evidence of significant cell death was observed at these exposure times. When NT2/D1 cells were exposed to curcuminoids for 2 h, some limited cell death was observed and the recovery of normal cell division was delayed to 5–7 days after exposure (Fig. 11A). The transient appearance of a larger cell type reminiscent of the morphology illustrated in Fig. 4 was also apparent during the first two days after curcuminoid exposure (not shown).

Significant cell death occurred within the first 24 h after exposure curcuminoids for 4 h. The cell number stabilized at a level of about 5% of the original value by 2 days after exposure (Fig 11B). During the following days some cell division resumed. However, the dividing cells did not immediately assume the phenotype of untreated cells. Instead, they were larger and they migrated to distribute themselves more evenly over the available growth surface (Fig. 11B, day 5, arrows). Indeed some of these cells further enlarged to assume what was reminiscent of the senescent phenotype (arrowheads) described in Fig. 4. On day 6 after exposure, some of the dividing cells showed evidence of reverting to the untreated phenotype. They were smaller and no longer mixed with the surrounding cell population (brackets). These cells became more numerous on day 7 and day 8 and formed rapidly dividing colonies with defined boundaries that expanded by crowding out existing populations of larger cells. During the next few days these cells completely overgrew the culture dish.

These results suggest that the same cellular effects that were obtained by chronically exposing NT2/D1 cells to lower concentrations of curcuminoids, could also be obtained by exposing cells transiently to a higher curcuminoid concentration. The main difference being that the effects were temporary and, with the exception of induced cell death and terminal differentiation, a subpopulation of cells recovered to their original state. The amount of time required for recovery was dependent on the curcuminoid exposure time.

## Discussion

The human embryonal carcinoma cell line NT2/D1 is a single-cell clone derived in multiple steps from a parental line designated as Tera-2 [25]. NT2/D1 cells are considered pluripotent and are in that respect similar to embryonic stem cells [26]. In contrast to stem cells, these cells are malignant and they contain karyotype abnormalities including polyploidy and chromosomal translocations [25]. However, similar rearrangements are also to varying degree found in most other cancer-derived cell lines. In addition, proteomic comparison of the cell membrane composition between embryonic stem cells and NT2/D1 cells revealed unique features, but also extensive similarities [26]. For comparative purposes with respect to the effects of curcuminoids, NT2/D1 cells should be considered a cancer cell line with some features in common with normal embryonic cells.

The effect of curcumin on cell division has been the subject of extensive studies with numerous cell lines and primary cells [27]. Curcumin induces growth arrest at various phases of the cell cycle, including G1/S, S, and G2/M. This is accomplished by modulating signal transduction pathways, including those regulated by e.g. NFκB [22], [23], [28], [29], [30], [31], [32], [33], [34], [35], [36], [37], Akt [33], [36], [38], [39], and NrF2 [40], [41], [42], [43], [44], [45]. These in turn affect the expression of gene products such as among others COX-2, cyclins, Bcl-2, and p53, thereby ultimately reducing or abolishing cell division (for reviews and additional references: [46], [47], [48], [49], [50], [51], [52]). The results presented here are qualitatively consistent with those observations. Curcuminoids diminish NT2/D1 cell division in a dose dependent manner upon repeated exposure to curcuminoids at 1–5 µM initial concentrations (Fig. 2A). Reports from other studies on the inhibition of cell division or cell survival following curcumin exposure vary over a wide curcumin concentration range. In cases where IC_50_ values for cell proliferation or viablility were determined for a variety of cancer cell lines, these ranged from 2.5–170 µM after curcumin exposure times of 24–120 h. Comparative values for primary cells ranged from 2.8 to >1000 µM (Table 2). However, in most reports curcumin was added as a single initial dose and not replenished before the time of analysis. It is therefore difficult to compare those studies with the results reported here, where curcuminoids were regularly replenished every 24 h. Indeed, comparable IC_50_ values for NT2/D1 proliferation would be about 2.3 µM after 96 h or 3.8 µM after 48 h of exposure. These values would likely be higher after a single application of curcuminoids, since these would be depleted after 24 h (Fig. 5). However, it is clear that different cell lines vary in their sensitivity to curcumin. For example, HeLa cells can be repeatedly exposed to more than 10 µM curcuminoid concentrations without apparent cell death under the same conditions as those applied here [13] (Table 1). The reasons for these different responses remain to be determined, but they may be related to the configuration of cellular sites available for curcuminoid binding and their role in the regulation of cell division. Although the effects of curcuminoids on normal cells were not examined in this study, it may be noted that a number primary cells from different tissues are sensitive to the effects of curcumin, albeit at generally higher concentrations than is typically the case for cancer-derived cell lines (Table 2). Furthermore, curcumin inhibits the proliferation of human retinal endothelial cells at concentrations of 1–30 µM [53]. Curcumin also inhibits cell division in mouse oocytes and early embryonic development at concentrations of <50 µM [54]. In a different study, significant apoptosis and inhibition of cell division was reported at the mouse blastocyst stage at a 24 µM curcumin concentration [55]. Survival of zebra fish embryos and larvae was also impaired after a 24 h exposure to curcumin with respective LD_50_-values of 7.5 µM and 5 µM [56]. These results suggest that the effects of curcumin on normal cells may not be as benign as generally presumed.

**Table 2:** Curcumin effect on cell proliferation.

CELLS^1^	TIME^2^ (H)	IC_50_ (µM)	REFERENCE
M21	48	19.05	[93]
SP6.5		22.39	
B-NHL	36	24.1	[94]
SK-NEP-1	48	4	[95]
PBMC*	48	2.8*	[96]
HeLa	48	14.32	[97]
Leiomyoma	48	10–15	[98]
Myometrial*		40*	
DLD-1	72	23.88	[99]
HCT-116		7.95	
SW480		6.49	
K562	48	41.89, 54.3	[100], [101]
MCF-7	n.s.	10.44	[102]
NCI-H460		20.6	
DU-145		27.6	
HepG2	48, 24	15.8, 13.95	[103]–[106]
MCF-7	48	24.8, 40	
MDA-MB-231	48, n.s.	11, 30	
Hs-68*	48	>50*	
C32	96–120	6.1–7.7	[107]
G-361			
WM266-4			
Osteosarcoma cell lines (6)	72	14.4–24.6	[108]
HF4.9	48	11.3	[109]
HF1A3		9.8	
HF28RA		7.5	
BT16A204G401	72	8.126.45.21	[110]
22rv1	24	44	[66]
LNCaP		48	
DU145		170	
PC-3		115	
Pancreatic Carcinomacell lines (6)	72	2.5–37.8**	[29]
Colon cancercell lines (3)	72	10.21–13.31	[111]
WI-38*		48.38*	
Pancreatic carcinomacell lines (5)	72	5.6–20.35	[112]
Bladder cells*		>1000*	

1 Assorted cell lines and primary cells.

2 Exposure time to curcumin before analysis.

* Primary cells, **Liposomal formulation, n.s.-not specified.

At higher initial curcuminoid concentrations (>8 µM), extensive NT2/D1 cell death occurs within two days of exposure (Fig. 2). At 46–49 µM curcuminoid concentrations, cell death is largely due to apoptosis via the activation of caspase 3/7 by both extrinsic (caspase 8) and intrinsic pathways (caspase 9). In this respect, these results are consistent with those reported for other cell lines at 20–100 µM curcumin concentrations [36], [57], [58], [59], [60] and for mouse embryos at 6–24 µM concentrations [55]. However, in NT2/D1 cells there is also a generalized activation of caspases in response to higher levels of curcuminoids (Fig. 4). This includes caspases 1,4 and 5, which are activated by extracellular inflammatory stimuli [61]. Caspase 2 is considered to be another initiator caspase whose function remains undetermined, whereas caspase 10 is functionally related to caspase 8. Finally, caspase 6 is an effector caspase with a different substrate specificity from caspases 3/7 [62]. Although at higher curcuminoid concentrations there is a universal activation of caspases in NT2/D1 cells, the dose-dependent cellular response to curcuminoids suggests that caspase activation may be similarly differentially regulated. In particular since caspases also have a function in cellular proliferation and differentiation [63], [64]. Although caspase-dependent apoptosis is a prominent feature at high curcuminoid concentrations, alternative mechanisms of cell death such as autophagy [38], [65], [66] or other caspase-independent pathways [67], [68] have been described.

Despite structural evidence of apoptosis and caspase activation, nucleosomal DNA fragmentation could not be demonstrated in NT2/D1 cells exposed to curcuminoids. In contrast, apoptosis induction by camptothecin did show concomitant DNA fragmentation indicating that this result was not due to methodological reasons. This phenomenon may indeed be cell-type specific since variable induction of DNA fragmentation was observed in leukemia and fibroblastic cell lines [69]. Lack of DNA fragmentation was also observed in Jurkat cells and and primary T-cells. This was interpreted as the direct inhibition of the CAD endonuclease by the binding of curcumin to the active site [58], [60]. However, CAD activation by caspase 3 occurs in the nucleus [70] and significant nuclear localization of curcumin has generally not been observed by histological methods [22], [23], [66], [71]. Nevertheless, at least one study has reported some degree of nuclear localization both by fluorescence microscopy and differential centrifugation [72]. It is therefore conceivable that a sufficient amount of curcuminoids are present in the nucleus to achieve CAD inhibition, or that under apoptotic conditions there is a different distribution of curcuminoids within cells due to altered exposure of subcellular compartments.

At intermediate curcuminoid concentrations (6–7 µM) a limited degree of cell death is observed and the remaining NT2/D1 cells gradually develop a phenotype that is consistent with senescence (Fig. 3). Cellular senescence is often induced by external stress signals such as hypoxia or by the action of cytokines [73], [74]. These in turn induce the production of reactive oxygen species either at the cytoplasmic level or in the mitochondrial transport chain. In particular in human cells, the activation of the p16INK4A/rb signaling pathway leading to the irreversible cessation of cell division has been implicated in the induction of senescence [75], [76], [77]. The observation that only a subpopulation of cells comprising about 40–50% of the original number (Fig. 2) developed into a senescent phenotype (Fig. 3), suggests that this may not be a random process. Since NT2/D1 cells are not homogeneous in terms of chromosome distribution, it is conceivable that some of the cells express biomarkers that make them more susceptible to cell death upon exposure to intermediate curcuminoid concentrations. In addition, the initial change in morphology in the remaining cells (Fig. 3) may indicate a form of cellular differentiation, which is consistent with the notion that this cell line can be induced to differentiate into various ectodermal cell types [14]. This concept may be extended to other cancer cell lines. For example, at intermediate curcuminoid concentrations a majority CCF-STTG1 cells showed robust differentiation into a phenotype resembling astrocytes before progressing to senescence. In contrast, only a minority of HeLa cells exhibited similar signs of differentiation and senescence (not shown). These variable effects among dedifferentiated cancer cell lines may again reflect the distribution of cells within the total population with biomarker profiles that designate them either for cell death or differentiation at intermediate curcuminoid concentrations.

There is a concentration-dependent gradient of biological effects of curcuminoids on NT2/D1 cells ranging from a decrease in the rate of cell division to cell death by apoptosis. Since most of these events can be accounted for by the interaction of extracellular signals on cell membrane receptors, it is not surprising that curcuminoids bind preferentially to membrane-associated fractions (Fig. 6). Although it has not been proven that the cell membrane is the primary target for curcuminoid binding, it is reasonable to assume that it is the initial target, in particular, in view of the rapid dissociation of non-metabolized curcuminoids from the cells following their removal from the medium (Fig. 8). Data based on fluorescence microscopy seem to suggest that curcumin also accumulates intracellularly [22], [23], [66]. Although fluorescence microscopic resolution does not appear to be sufficient to clearly differentiate between cell membrane and intracellular distribution, a study using confocal microscopy to determine curcumin localization concluded that curcumin almost exclusively partitioned into membrane structures [71]. A different study employing differential centrifugation also showed preferential binding to membrane fractions [72]. However, Kunwar et al. [72] used 0.6% Nonidet P-40 to disrupt cells. Even at such a concentration, this detergent substantially increases the solubility of curcumin, which is likely to dislodge membrane bound curcumin during extraction and fractionation. This would explain the significantly larger proportion of curcumin recovered from the cytoplasmic fraction in that study compared to the very low levels of cytoplasmic curcuminoids reported here (Fig. 6). Another study employed sonication as a method of cell disruption [78]. Cells were then centrifuged at 9,000×g and the supernatant was further centrifuged at 100,000×g. This resulted in total amounts of curcumin 2–3 times higher in the 100,000×g membrane pellet than in the cytosolic supernatant, although the amount bound to the initial 9,000×g pellet was not reported.

The cellular sites that are targets for curcuminoid binding remain largely unknown. However, they are likely to be numerous with varying individual binding affinities. Consequently, the overall K_D_ values for specific cellular binding of curcuminoids determined here are not to be considered as representing the affinity for a single binding site. Instead, they represent the sum of numerous binding sites with similar curcuminoid binding affinities. Protein components embedded in the membrane such as cellular receptors, a wide variety of which have been identified in NT2/D1 cells [26], seem to be likely targets. In particular, since curcumin has been shown to interact with proteins, including albumin and numerous mediators of cellular transduction (for references see: [79]). However, direct binding of curcuminoids to the exposed polar moieties of membrane lipids is also likely. This clearly has implications in the context of membrane compartmentalization into lipid rafts [80]. The binding of curcuminoids to such ordered lipid-protein assemblies could affect both signal transduction events and provide an avenue for internalization [81]. Indeed, lipid raft and receptor mediated endocytosis has been described for the cellular uptake of resveratrol, a biochemically and functionally related polyphenol [82].

The overall K_D_ for total specific binding of soluble curcuminoids to NT2/D1 cells was here determined to be in the 6–10 µM range using variable amounts of curcuminoid-saturated serum (Fig. 7). These K_D_ values are based on cellular binding versus the concentration of total soluble curcuminoids, which are primarily present as complexes with serum components under saturating conditions. Although this value is in the same range as the concentrations necessary to induce biological effects, actual cellular binding under the employed experimental conditions of variable curcuminoid and constant serum concentration would be considerably lower due to competition for cellular binding by free serum (Fig. 7). This also has therapeutic implications, since *in vivo* the lower degree of cellular curcuminoid binding in the presence of excess serum would further limit the cellular response at an already low level of bioavailability. The situation is further complicated by the observation that there are at least two different binding sites for curcumin on serum albumin [83], which is a primary curcuminoid-binding component in serum [13], [84]. Indeed, the stoichiometric ratio of curcuminoids/albumin at binding saturation suggests the presence of at least three potential albumin binding sites [13]. It is likely that the albumin-bound curcuminoids are released sequentially for binding to cellular receptors. It is also plausible that the serum- or albumin-bound curcuminoids occupy different binding sites, depending on whether they are SOLID- or DMSO-solubilized. Upon mixing such saturated preparations, intermolecular rearrangements could occur leading to a redistribution of curcuminoid binding sites within the serum that would have a similar competitive effect as adding curcuminoid-free serum. This would then result in an increase in the overall apparent K_D_ for cellular binding as was observed for albumin and serum solutions (Fig. 7). Consequently, differences in apparent K_D_s for cellular binding resulting from using different soluble curcuminoid preparations would not represent variable affinities for cellular receptors, but rather varying affinities for curcuminoid-binding to serum components.

A comparison of the cell lines NT2/D1 (embryonal carcinoma), HeLa (cervical carcinoma), and CCF-STTG1 (astrocytoma), shows that these cells differ in their rate of cell division, their affinities for curcuminoids, and their response to curcuminoids (Table 1). The cellular responses to curcuminoids in the form of cell death and senescence are related to the cellular affinities for curcuminoids. The reasons for these differences in cellular affinities for curcuminoids between cell lines are currently unclear. However, they are likely associated with the lipid and protein organization within the cell membranes of the different cell lines. As a result, the cellular affinity and response to endogenous or serum-derived growth factors may also vary, resulting in different rates of cell division.

Although differential effects of individual curcuminoids on a variety of biological responses have been reported (review: [2]), it did not matter in these experiments whether the curcuminoids solubilized in FCS contained either predominantly CUR (DMSO-solubilized) or BDMC (SOLID-solubilized). The effects on cell division, apoptosis, induction of senescence, or activation of caspases were indistinguishable for the two preparations within the limits of the established experimental parameters. This could be because the two preparations contained a complement of all curcuminoids, albeit at different relative abundance, and as such the experimental design did not allow for a sufficient resolution to detect differential effects. In addition, the lack of difference may be explained by the different properties of curcuminoids in solution and in terms of binding and metabolism. Although BDMC and DMC have higher apparent binding affinities for cellular receptors, they are also metabolized at a relatively faster rate, resulting in a faster depletion from cells and media (Figs 5 and 8). Conversely, over a 24 h period CUR is more readily subject to chemical decomposition than BDMC or DMC [13], [24], [85]. Overall, the sum of these effects may neutralize any differential biological effects of the individual curcuminoids in these preparations.

Curcuminoids bound to cell membrane receptors could act on signal transduction pathways by either blocking the access of growth factors present in the serum or by inducing cellular responses themselves. However, simply reducing or eliminating the serum in the media does not produce responses similar to those induced by curcuminoid exposure. Indeed, reducing serum concentrations to 1% merely resulted in a moderate decrease in the rate of cell division. Completely eliminating the serum induced extensive cell death within 24 hr while the induction of senescence could not be demonstrated at any serum concentration (not shown). Furthermore, exposing NT2/D1 cells to high concentrations of curcuminoids for shorter time periods of 0.5–4 h resulted in cellular responses similar to those obtained with chronic exposure at lower concentrations (Fig. 10). These observations suggest that curcuminoid-binding to cells activates signal transduction pathways that lead to dose- or time-dependent reductions in cell division, induction of senescence, or apoptosis. Indeed, curcumin has been identified as an activating ligand for the vitamin D receptor (VDR) [86], which is also highly expressed in NT2/D1 cells [87]. The intracellular VDR could potentially be accessed by curcuminoids via the same cell membrane receptor- or caveolae-mediated mechanisms that are also used to transport steroid hormones [88]. That curcuminoid binding *per se* is responsible for these effects is further exemplified by the observations that presumably inactive metabolites such as those described here (Fig. 9), and conjugated products such as curcumin sulfate (not shown) or glucuronidate [78] show no significant cellular binding. Similarly, upon incubating cell cultures with hexahydrocurcumin, the metabolite was found in the cytosolic but not in the membrane fraction [78], indicating a general loss of membrane binding upon curcumin reduction. It is indeed likely that the poor solubility of curcumin in water is a crucial property that forces it to be shuttled between amphophilic binding sites in serum components and the cellular binding sites, a property also shared by steroids and related compounds.

The primary metabolic reduction products of curcumin *in vivo*, in tissue slices, and in cell extracts are tetrahydrocurcumin and hexahydrocurcumin with smaller amounts of dihydrocurcumin and octahydrocurcumin, a conversion that takes place primarily in the liver and intestine [78], [89], [90]. Hexahydrocurcumin has also been identified in cultured Ishikawa and HepG2 cells [78]. In this study, hexahydrocurcuminoids were identidied as the major metabolites in both HeLa and CCF-STTG1 cells, while the latter also generated significant amounts of octahydrocurcuminoids (Fig. 10). However, none of those reduction products were identified in NT2/D1 cells. Instead, novel curcuminoid reduction products with the same molecular weight as tetrahydrocurcuminoids but with different spectral and chromatographic elution properties were detected (Fig. 9). The preliminary structure assignment of these compounds shows different positions of the remaining double bonds compared to tetrahydrocurcumin. The same conversion products were initially also found in the astrocytoma cell line CCF-STTG1 and in HeLa cells upon shorter incubation times (<5 h) and are thus not unique to NT2/D1 cells (not shown). This shows that at least in culture, an alternative reduction pathway is available to inactivate curcuminoids. Whether this also exists *in vivo* remains to be established. The enzymes responsible for the reductive conversion have not been identified, but at least *in vitro* it can be accomplished with alcohol dehydrogenase [89]. In any case, most reductases are cytoplasmic enzymes and consequently it would require the curcuminoids to be internalized after binding to the cell membrane. The resulting curcuminoid reduction products would then loose their membrane binding properties upon which they are expelled into the medium. That this is an efficient process is suggested by their lack of detection in cells.

In conclusion, this study has described the cellular binding and metabolic fate of curcuminoids. While focusing primarily on NT2/D1 cells as a model system, selected binding parameters and cellular effects were also compared to HeLa and CCF-STTG1 cells. For this purpose, commercial grade curcumin was first solubilized in FCS at saturating concentrations in the 1 mM range. As a result, maximal curcuminoid concentrations of about 50 µM can be achieved in culture media containing 5% total FCS. This method of solubilization is different from that typically employed in other studies, where curcumin is dissolved in an organic solvent and added at appropriate dilutions to the final culture medium. Directly solubilizing curcuminoids in serum offers several advantages over adding curcuminoids dissolved in organic solvents. These include the lack of initial precipitation, lower overall concentrations of organic solvents, and greater precision in achieving small increments of curcuminoid concentrations in the media. The lack of precipitation is particularly relevant to studies where curcuminoid concentrations in excess of 100 µM are employed with media containing 10% FCS. Since the vast majority of soluble curcuminoids are complexed with FCS, any excess beyond the saturation point will remain in the media as particulate matter with unknown consequences in terms of binding, metabolism and biological effects. Furthermore, commercial grade curcumin can be solubilized in serum by two procedures resulting in similar total concentrations but profoundly different final compositions. Direct mixing of such solutions at different ratios thus easily generates preparations with a range of curcuminoid profiles. This has allowed for the titration of their biological effects at curcuminoid concentration increments of about 1 µM in the media. Future projects will address the modulation of specific signal transduction pathways using the same range of curcuminoid concentrations that result in reduced rates of cell division, induction of senescence, and cell death. Based on such data, it should be possible to correlate specific biological responses with the incremental binding of curcuminoids to cells and specific signal transduction events. Given a sufficient resolution, this could help clarify the sometimes conflicting data that has emanated from the large body of literature encompassing curcumin research.

## Materials and Methods

### Standard Reagents and Solutions

Technical and analytical grade curcumin were obtained from the Cayman Chemical Company. Technical grade curcumin (>65%) contains all three curcuminoids whereas analytical grade curcumin is highly enriched in CUR (>90%) together with small amounts of DMC. Pure synthetic CUR was provided by Dr. Francis Johnson. DMSO [ACS reagent], BSA [Fraction V, 96–99% albumin] were obtained from Sigma Chemical Company. DMEM, 4.5 mM glucose [Lonza], 1-butanol (HPLC grade), Acetonitrile and water (both HPLC grade) were purchased from Fisher Scientific and Trypsin-EDTA (0.05%) from GIBCO. FCS was supplied by Aleken Biologicals. Tetrahydrocurcumin and octahydrocurcumin used as standards for reversed phase chromatography and mass spectrometry were obtained from the Sabinsa Corporation and hexahydrocurcumin from Sigma-Aldrich. All other standard laboratory chemicals were provided by Research Organics.

BSA was prepared as a 10% (w/v) stock solutions in PBS (137 mM NaCl, 10 mM Phosphate, 2.7 mM KCl, pH 7.4). Sera were thoroughly mixed and used as provided. Autoclaved isotonic (0.9% w/v) or hypotonic saline (0.6% w/v) solutions were prepared with deionized water. Butanol for curcuminoid extraction was equilibrated with an excess of deionized water containing 1 mM hydrochloric acid and used after phase separation.

### Curcuminoid Solubilization and Cell Culture Media Preparation

For cell culture media preparation, 25–50 mL of FCS was either mixed with solid curcuminoids (SOLID-solubilized, 50 mg/mL) or with 5 µl/mL of curcuminoids predissolved in DMSO at a 1 M concentration (DMSO-solubilized). In both cases, the suspended curcuminoids were stirred in 50 mL Erlenmeyer flasks at 4°C for 16–24 h. Residual insoluble curcuminoids were removed by two successive centrifugations at 20,000×g followed by sterilization through 0.45 µm filters [13]. In some procedures, the SOLID- and DMSO-solubilized curcuminoid-saturated FCS was mixed at a ratio of 1∶1 (SOLID+DMSO). Curcuminoid-containing media were diluted with standard medium (DMEM supplemented with 5% FCS) to obtain the desired final curcuminoid concentrations.

### The Effects of Curcuminoids on Cell Division and Survival

NT2/D1 cells (ATCC#: CRL-1973) were grown in 5.6 mL standard medium at 37°C in an incubator equilibrated with 5% CO_2_. To investigate the effect of increasing curcuminoid concentrations on cell division and survival, cells were seeded at about 20% confluence (∼1.2×10^6^ cells) in 25 cm^2^ flasks (Sarstedt). The following day (day 0), the standard medium was replaced with media containing 1–10 µM SOLID- or DMSO-solubilized curcuminoids. HeLa (ATCC#: CCL-2) and CCF-STTG1 (ATCC#: CRL-1718) cells were similarly incubated, albeit at higher (10–30 µM) curcuminoid concentrations due to the reduced curcuminoid sensitivities of these cell lines.

In instances where NT2/D1 cells were transiently exposed to higher curcuminoid (SOLID+DMSO) concentrations, the standard medium was replaced with curcuminoid-containing media (47 µM) on the day after seeding (day 0) and incubated for 0.5 h, 1 h, 2h, and 4 h. Thereafter, the curcuminoid-containing medium was removed and the cells washed twice with 6 mL of standard medium followed by incubation for 1–8 days in 6 mL of standard medium. In all cases, the media were changed every 24 h and the cells photographed and counted for 1–8 days. If necessary, cells were diluted and subcultured at a ratio of 1∶5 upon reaching confluence.

### Reversed Phase Chromatography

Curcuminoids were extracted from cells and media with acidified water-saturated butanol. The butanol was evaporated in a Savant Speed-Vac concentrator attached to a vacuum pump. The dried residue was reconstituted in a 0.2–3.6 mL solution (FPLC loading buffer) containing 75% eluent A (5% acetonitrile, 0.01% ammonium acetate, pH 4.5, and 95% water) and 25% eluent B (95% acetonitrile, 0.01% ammonium acetate, pH 4.5, and 5% water), yielding a final acetonitrile concentration of 27.5%. Reversed phase chromatography was performed on an FPLC ÄKTA Purifier system equipped with a Source 5RPC ST 4.6/150 column (GE Healthcare). Reconstituted curcuminoids (0.2–3.6 mL) were loaded onto a 0.1 mL sample loading loop and separated with a 35 mL linear gradient, which ranged from a starting concentration of 75% eluent A and 25% eluent B to a final concentration of 15% eluent A and 85% eluent B (27.5%–81.5% acetonitrile) at a flow rate of 1 mL/min. The eluent was monitored at wavelengths of 280, 310, and 427 nm. Quantitation of individual peaks was carried out with the Unicorn peak integration program (version 5.10).

### Cell Numbers, Concentration Calculations, and Statistical Analysis

NT2/D1 cells were photographed in three random viewing fields using phase contrast microscopy and manually counted. Alternatively, a dose curve was created that correlated cell number and total protein content. Linearity extended up to 10^7^ cells/25 cm^2^ flask with 1 mg of protein representing 6.35×10^6^ cells (not shown).

Curcuminoids were separated by reversed phase chromatography and quantitated based on a standard curve for curcumin (analytical grade) by integration of the peaks with the FPLC Unicorn (version 5.10) program (Pharmacia-GE). The molar absorptivities (ε) of the three curcuminoids dissolved in ethanol were previously shown to range from 6.73 (×10^4^ L cm^–1^mol^–1^) for CUR, 5.78 for DMC and 4.95 for BDMC at 425 nm [91]. Since the molar absorptivity for curcumin dissolved in butanol was essentially the same as in ethanol (not shown), the integrated values of the DMC and BDMC peaks were multiplied by the respective factors 1.17 and 1.36 to obtain molar concentrations in all experiments, except for those relating to the identification and quantitation of curcuminoid metabolites. In those cases, the area under the peaks was used directly for comparative purposes.

Unless otherwise indicated, all data points were calculated as the average of 3–10 independent experiments, each assayed in duplicate. Error bars represent the standard deviation from the average. In the case of dose curves, individual data points were combined from at least three separate experiments and the integrity of the data is indicated by the associated R^2^-values of the curve fit (Sigmaplot, version 11).

### Caspase and DNA Fragmentation Assays

For the determination of caspase activities, NT2/D1 cells were grown in 75 cm^2^ flasks to 90% confluence (∼2×10^7^ cells). Cells were then either harvested directly (time 0 h) or exposed to 46–49 µM curcuminoids in 5% FCS in a total volume of 12 mL for 6, 12, or 18 h. Cells were scraped directly into the incubation media and centrifuged at 5,000×g for 5 min. The pellets were resuspended in 1 mL of PBS, transferred to 1.5 mL microfuge tubes and centrifuged at 12,000×g for 1 min. The pellet was washed twice with 1 mL of PBS. The final pellet was assayed for caspase activity with a colorimetric assay kit (BioVision). Each pellet was resuspended in 300 µl cell lysis buffer and left on ice for 5 min. The lysed cells were centrifuged for 10 min at 14,000×g at 4°C to pellet nuclei and membranes. The supernatants were carefully removed and transferred to fresh microfuge tubes. For caspase assays, the supernatants were aliquoted in volumes of 50 µL to 5 fresh microfuge tubes containing either 1 µL DMSO (blank) or 1 µL of either specific or universal irreversible caspase inhibitor (5 mM stock in DMSO). Solutions were gently mixed and left at 20°C for 5 min. Thereafter, 50 µL of 2× reaction buffer supplemented with 10 mM dithiothreitol (1 M stock solution) was added to each tube. Tubes designated to provide background absorption received 2× reaction buffer without substrate and the remaining tubes received 2× reaction buffer containing paranitroanilin (pNA)-caspase substrate premixed at a 200 µM concentration. Tubes were mixed by gentle vortexing and incubated at 37°C for 2 h. Thereafter, 120 µL of dilution buffer was added to each tube and aliquots of 200 µl were read at 405 nm in a microplate reader (Bio-Tek Instruments). Substrate-specific caspase activity was defined the value of total pNA conversion obtained from samples without caspase inhibitor subtracted from values obtained from samples containing caspase inhibitor.

The pNA-substrates considered specific for individual caspases (obtained from BioVision or AnaSpec) are listed in Fig. 3. Specific caspase inhibitors used were caspase-1 inhibitor II (AnaSpec), caspase-2 inhibitor I (Calbiochem) and Z-VDVAD-FMK (BioVision), caspase-3 inhibitor I and II, caspase-3/7 inhibitor II, caspase-4 inhibitor I, caspase-5 inhibitor I, caspase-6 inhibitor I, caspase-8 inhibitor II, caspase-9 inhibitor I and III (all Calbiochem), and caspase-10 inhibitor AEVD-FMK (BioVision). The universal caspase inhibitors used were caspase inhibitors III and VI (Calbiochem).

For nuclear DNA fragmentation assays, NT2/D1 cells were grown to 90% confluence in 25 cm^2^ flasks (∼6×10^6^ cells/flask). Cells were then either harvested directly (control, time 0 min) or exposed to 46–49 µM curcuminoids in DMEM with 5% FCS in a total volume of 5 mL for 6, 12, or 18 hr, or cells were exposed to 5 µM camptothecin (BioVision) in the same medium for 6 hr. Cells were trypsinized and processed with the Quick Apoptotic Ladder Detection Kit (BioVision) according to the manufacturer’s instructions except that the recommended volumes were doubled to account for the higher cell counts. Ethidium bromide stained gels were UV-transilluminated and photographed.

### SA-β-galactosidase Staining

NT2/D1 cells were grown in 25 cm^2^ flasks with standard medium to about 70% confluence. Thereafter, cultures were incubated in media containing 6 µM of SOLID- or DMSO-solubilized curcuminoids that were changed every 24 h. Cultures were stained for SA-β-galactosidase after 4 and 12 days in culture essentially as described elsewhere [20], [92]. Briefly, cells were washed twice with 5 mL PBS (pH 7.4) and then fixed with 5 mL 0.5% glutaraldehyde in PBS (pH 7.4). Thereafter, cells were washed 6 times with 5 mL PBS (pH 6). Finally, 40 µL of X-gal (5-Bromo-4chloro-3-indolyl-b-D-galactoside, 50 mg/mL in N,N-dimethylformamide) was added to 4 mL of PBS (pH 6) containing 5 mM potassium ferricyanide and 5 mM potassium ferrocyanide. Cells were incubated in that solution for 6 h at 37°C without CO_2_. Control cultures without curcuminoids were similarly processed. Cells were viewed and photographed under phase contrast with a Nikon Eclipse TE300 inverted microscope.

### Cellular Curcuminoid Binding and Media Concentrations

NT2/D1 cells were grown in 25 cm^2^ flasks to a density of about 90% confluence in standard medium. To establish a time course for cellular binding of curcuminoids, cells were incubated for 5 h with 5.6 mL of medium containing serum-solubilized curcuminoids (SOLID+DMSO, 1∶1) diluted with standard medium to achieve a final 27 µM concentration. At each indicated time point, aliquots of 0.4 mL were taken from the media for butanol extraction. The remaining media were aspirated and the cells were washed three times with 5 mL of 0.9% NaCl. The cells were then scraped into 1 mL of 0.9% NaCl and centrifuged at 2,000×g for 1 min. The pellet was resuspended in 1 mL of 0.6% NaCl and aliquots of 10 µl were taken for protein determination. The cell suspension was again centrifuged and the final pellet was suspended in 0.4 mL of 0.6% NaCl and extracted with 0.8 mL butanol. Cells and media were similarly processed to determine changes in curcuminoid levels over a 24 h period except that the incubation medium contained a starting concentration of 47 µM curcuminoids (SOLID+DMSO, 1∶1).

For curcuminoid dose curves to determine binding dissociation constants with variable serum concentration, media were prepared with increasing amounts (20 µL-6 mL) of curcuminoid-solubilized serum and DMEM for a total volume of 6 mL. From these media, 0.4 mL was removed for butanol extraction. Cells were then incubated with the remaining 5.6 mL of media for 1 hr. For curcuminoid dose curves at constant serum concentrations, increasing amounts of serum-solubilized curcuminoids were mixed with regular serum for constant total serum concentration of 5%, 50%, or 100% and diluted with DMEM in a total volume of 6 mL. Media for dose curves to determine cellular binding with a constant amount of serum-solubilized curcuminoids (5%) and increasing amounts of regular serum were similarly prepared with DMEM. Cells and media were then collected and processed as described above.

To determine the rate of dissociation of cellular-bound curcuminoids, cells were first incubated with complete medium containing 47 µM curcuminoids (SOLID+DMSO, 1∶1) for 1 h. The cells were then washed 3× with 5 mL of PBS and further incubated with 2 mL of DMEM medium with or without 5% FCS for another 2 h. At the indicated time points, the entire media volumes were divided into five fractions of 0.4 mL and each extracted with 0.8 mL butanol. The media fractions were evaporated under vacuum and combined in 0.2 mL of FPLC loading buffer for reversed phase chromatography. Cells were harvested and processed as described above.

When the dissociation of cellular-bound curcuminoids was examined under cell-free conditions, cells were first washed 2× with 5 mL of PBS and then briefly with 5 mL of hypotonic 10 mM hepes (pH 7). Cells were then incubated with 2 mL of 10 mM hepes, pH 7. This resulted in the rupture and detachment of cells. At the indicated time points, the suspension of cell fragments was collected and centrifuged at 18,000×g for 10 min. The supernatants and pellets were processed as described above.

### Detection of Metabolic Products

Metabolic products of curcuminoids were detected in incubation media at 310 nm wavelength during reversed phase chromatography. Metabolic products were quantitated by incubating NT2/D1 cells (90% confluence in 25 cm^2^ flasks) for 5 h in 8 ml of media containing either 47 µM curcuminoids (SOLID+DMSO, 1∶1) in 5% FCS, 24 µM curcuminoids in 2.5% FCS, or 24 µM curcuminoids in 5% serum. Aliquots of 0.4 ml of media were removed at indicated time points, extracted with 0.8 mL of butanol and processed as described. After reversed phase chromatography, the areas under the peaks were adjusted for media volume and plotted against time. At the end of the incubation period, cells were harvested and processed as described for curcuminoid binding.

For mass spectrometry of the CUR metabolic product, a 25 cm^2^ flask with NT2/D1 cells at 90% confluence was incubated for 4 h with 4 mL of DMEM medium containing 50 µM analytical grade curcumin in 5% FCS. Ten aliquots of 0.4 mL were extracted with 0.8 mL of butanol and dried under vacuum. The dried residues were each solubilized in 0.2 mL FPLC loading buffer and separated by reversed phase chromatography. Absorption peaks at 310 nm wavelength that corresponded to the CUR metabolic product were collected and dried. The peak fractions were sequentially dissolved in a total volume of 0.2 mL FPLC loading buffer and again separated by chromatography. The metabolic fraction of curcumin was this time collected as a single fraction. Peaks containing CUR, Octahydrocurcumin, and tetrahydrocurcumin were similarly separated, collected and used as standards for comparison. The collected fractions were again dried and resolubilized in 0.1 mL of 50% acetonitrile. Aliquots were further diluted with the same solution at a ratio of 1∶5 and analyzed in Thermo TSQ Quantum Access Triple Quadrupole and LTQ Ion Trap mass spectrometers (Thermo-Fisher) at a flow rate of 5 µL/min in negative ion mode.

To compare metabolic products generated by different cell lines, NT2/D1, HeLa, and CCF-STTG1 cells (90% confluence in 25 cm^2^ flasks) were incubated in 4 ml of media containing 47 µM curcuminoids (SOLID+DMSO, 1∶1). After 24 h, 0.4 ml aliquots of media were butanol extracted and analyzed by reversed phase chromatography at wavelengths of 280 nm and 310 nm. Octa- and hexahydrocurcuminoids were identified by co-elution with commercial standards and mass spectrometry.

### Cell Fractionation

NT2/D1 cells were grown to 90% confluence in two 175 cm^2^ flasks (∼4×10^7^ cells) and incubated with 5% curcuminoid-saturated serum (47 µM; SOLID+DMSO, 1∶1) for 1 hr. Cells were first rinsed 3× with 25 mL and then scraped into 10 mL 0.9% NaCl, combined, and centrifuged at 5,000×g. The cell pellet was suspended in 2 mL PBS (pH 6.8), transferred to a microcentrifuge tube, and recentrifuged. The resulting pellet was rapidly washed with 1 mL of 50 mM Tris, pH 6.8 and recentrifuged. The final pellet was suspended in a total volume of 1.6 mL of 10 mM Tris, pH 6.8 and divided into four equal parts. One part representing the total cell content of curcuminoids, was directly extracted with butanol. The remaining three parts (0.4 mL each) were disrupted by three different procedures. One part was homogenized in a 1 mL glass homogenizer (Wheaton) with a tight pestle (20 strokes). To another part, 0.1 g of glass beads (Sigma, 425–600 µm) was added. This was followed by 3 freeze-thaw cycles with vortexing during the thaw-phase. A third part was placed in ice water and sonicated 3× at full power (Ultrasonic Systems Inc., model 250) for 5 seconds each. Thereafter, 0.4 mL of 500 mM sucrose, 50 mM Tris, pH 6.8, and 2.5 mM MgCl_2_ was added to each part. The samples were then sequentially centrifuged at 1,000×g, 15,000×g, and 100,000×g (Fig. 6A). The pellets after the first two centrifugations were washed once in 1 mL of PBS (pH 6.8) and recentrifuged. The resulting pellets were resuspended in 0.4 mL PBS (pH 6.8) and extracted with 0.8 mL of butanol. After the final 100,000×g centrifugation, the entire supernatants (0.6–0.7 mL) were extracted twice with 0.6 mL of butanol and the extracts combined. The final pellet was directly resuspended in 0.4 mL of 0.6% NaCl and extracted with 0.8 mL of butanol. Before each extraction and after each step of the procedure, aliquots of 3×10 µL were withdrawn for protein determination (BCA Protein Assay, Thermo Scientific) with a mixture of RNase, Lysozyme and BSA (equal parts by weight) as a reference standard. All butanol extracts were evaporated under vacuum and the dry residues solubilized in 0.2 mL loading buffer for reversed phase chromatography.
